# ECHO-liveFISH: *in vivo* RNA labeling reveals dynamic regulation of nuclear RNA foci in living tissues

**DOI:** 10.1093/nar/gkv614

**Published:** 2015-06-22

**Authors:** Ikumi Oomoto, Asuka Suzuki-Hirano, Hiroki Umeshima, Yong-Woon Han, Hiroyuki Yanagisawa, Peter Carlton, Yoshie Harada, Mineko Kengaku, Akimitsu Okamoto, Tomomi Shimogori, Dan Ohtan Wang

**Affiliations:** 1 Institute for Integrated Cell-Material Sciences (WPI-iCeMS), Kyoto University, Yoshida-Honmachi, Sakyo-ku, Kyoto 606-8501, Japan; 2 Graduate School of Biostudies, Kyoto University, Yoshida-Honmachi, Sakyo-ku, Kyoto 606-8501, Japan; 3 Brain Science Institute, RIKEN, Hirosawa, Wako City, Saitama 351-0198, Japan; 4 JSPS Research fellow, Japan Society of Promotion of Science, 5-3-1 Kojimachi, Chiyoda-ku,Tokyo 102-0083, Japan; 5 Advanced Science Institute, RIKEN, Hirosawa, Wako City, Saitama 351-0198, Japan; 6 Research Center for Advanced Science and Technology, University of Tokyo, Komaba, Meguro-ku, Tokyo 153-8904, Japan

## Abstract

Elucidating the dynamic organization of nuclear RNA foci is important for understanding and manipulating these functional sites of gene expression in both physiological and pathological states. However, such studies have been difficult to establish *in vivo* as a result of the absence of suitable RNA imaging methods. Here, we describe a high-resolution fluorescence RNA imaging method, ECHO-liveFISH, to label endogenous nuclear RNA in living mice and chicks. Upon *in vivo* electroporation, exciton-controlled sequence-specific oligonucleotide probes revealed focally concentrated endogenous 28S rRNA and U3 snoRNA at nucleoli and poly(A) RNA at nuclear speckles. Time-lapse imaging reveals steady-state stability of these RNA foci and dynamic dissipation of 28S rRNA concentrations upon polymerase I inhibition in native brain tissue. Confirming the validity of this technique in a physiological context, the *in vivo* RNA labeling did not interfere with the function of target RNA nor cause noticeable cytotoxicity or perturbation of cellular behavior.

## INTRODUCTION

The concentration of RNA–protein complexes within specific subcellular foci is a commonly used strategy to control gene expression in response to environmental cues in eukaryotic organisms. This spatial regulation of intranuclear RNA foci plays a fundamental role in genome regulation, RNA processing as well as ribosome biogenesis (1–8). Furthermore, ribonuclear foci that contain abnormally lengthened repeat sequences act as pathological sites in the dysfunction and degeneration of muscular and nervous tissues in human diseases (9,10). Understanding the dynamic regulation of intranuclear RNA foci is therefore an important step toward manipulating these functional sites in both physiological and pathological contexts.

Given the abundance of local concentrations of RNA and proteins, many subnuclear domains such as nucleoli, splicing speckles, paraspeckles, Cajal bodies, histone locus bodies and promyelocytic leukemia bodies have been readily identified by *in situ* hybridization, immunostaining and exogenous labeling techniques combined with fluorescence microscopy (11,12). Time-lapse imaging with fluorescent protein tags has demonstrated high mobility and dynamic shuttling of many protein components between the nuclear bodies and nucleoplasm (13). In contrast, the other major component of nuclear bodies, RNA, has rarely been studied in the context of its nuclear dynamics due to the lack of effective imaging methods. Recently developed live-cell imaging methods have made it possible to visualize the dynamics of a single, cytoplasmic, mRNA molecule in a living mouse (14,15). Examples include molecular beacons used in developing *Drosophila* embryos that have successfully enabled visualization of the accumulation of *oskar* mRNA to the posterior pole (16,17) and the use of *in vivo* injection of fluorescently labeled mRNAs into the syncytial blastoderm of flies which, revealed motor-dependent apical localization of hairy transcripts (18). These methods are highly advantageous not only because they allow endogenous RNA to be monitored in its native context but also provide an opportunity to correlate RNA dynamics with physiological and behavioral stimuli. However, none of the current methods have been, or can be, readily applied to studying the dynamics of intranuclear RNA *in vivo*. Techniques that allow for *in vivo* RNA imaging would provide a necessary complement of the studies of target cytoplasmic RNA.

To establish an effective *in vivo* RNA imaging method a labeling technique with a high signal-to-noise ratio is essential. Previously, we devised an RNA detection method using exciton-controlled hybridization-sensitive fluorescent oligonucleotide (ECHO) probe technology and successfully established a wash-free fluorescence *in situ* hybridization (ECHO-FISH) method to detect specific RNA transcripts in fixed cells (19). In this method we have used linear deoxyoligonucleotide probes (13–50 nt) labeled with a pair of thiazole orange (TO) dye moieties, TO has an excitation maximum at 514 nm (D514; (20). When single-stranded, D514 containing probes do not emit fluorescence due to the excitonic coupling between the TO dye moieties. Upon hybridization to a complementary sequence, the combined effect of breaking the excitonic coupling and intercalation of TO into the probe-RNA duplex leads to a drastic increase in fluorescence emission (21,22). ECHO-FISH alleviates the time and labor present in conventional FISH protocols without compromising detection stringency. However, its usefulness has been limited to *in vitro* studies due to the lack of effective delivery as well as high-resolution imaging methods for monitoring fine intranuclear foci *in vivo*. Moreover, cytotoxicity and potential influence on the metabolism and function of the target RNAs in a living organism has yet to be carefully evaluated in order to understand the physiological relevance of results produced using ECHO-FISH.

Herein, we describe a live-cell imaging method for monitoring endogenous RNA intranuclear foci labeled with ECHO probes, in both cultured cells and complex living tissue. We use an *in vivo* electroporation technique to effectively deliver ECHO probes into living animals and perform high-resolution time-lapse imaging in acute brain slices to monitor the spatiotemporal dynamics of single RNA intranuclear foci. We are able to confirm labeling specificity *in vivo* with using the specific localization patterns of the target RNA. We observe spatial constraints of nuclear RNA foci, which can be dynamically regulated by cellular transcriptional activity. Notably, *in vivo* labeling neither interferes with the function of the target RNA nor causes detectable cytotoxicity or perturbation of cellular behavior, validating the physiological relevance of this technique.

## MATERIALS AND METHODS

### Animals

ICR mice were purchased (Shimizu, Japan) and housed at iCeMS animal facility. This study was carried out in accordance with the Guide for the Care and Use of Laboratory Animals from the Society for Neuroscience and was authorized by the Animal Care and Use Committee of Kyoto University. *Gallus gallus* eggs were purchased (Yamagishi, Japan) and incubated at 38°C in the humidified atmosphere.

### Reagents

0.1% gelatin (Millipore, ES-006-B), actinomycin D (Wako, 018-21264), Alexa Fluor 633 Goat Anti-Mouse IgG (H + L) (life technologies, A-21050), anti-DsRed2 antibody (used at 1:1000 for immunostaining, Clontech, 632496), anti-Puromycin antibody (used at 1:1500 for immunostaining or 1:12500 for western blot, Millipore, clone 4G11), B27 supplement (Gibco, 17504-044), bovine serum albumin (Sigma, A3294), CC/Mount™ (Diagnostic BioSystems, K 002), cycloheximide (Nakalai, 66-81-9), cisplatin (Wako, 033-20091), Dulbecco's modified Eagle's medium (DMEM) (Gibco, 12320-032), ECL Prime (GE Healthcare, 72-AS01-20), fetal bovine serum (FBS) (Gibco, 10437-028), GlutaMAX (Gibco, 35050-061), lipofectamine2000 Transfection Reagent (Invitrogen, 11668-019), methotrexate (Wako, 139-13571), neurobasal-A medium (Gibco,10888-022), oxaliplatin (Wako, 156-02691), paraformaldehyde (Sigma, P6148), penicillin-streptomycin (Sigma, P4333), ProLongGold Antifade Reagent with DAPI (Molecular Probes, P-36931), protease inhibitor cocktail (Sigma, P8340), puromycin (Sigma, P8833), RPMI 1640 media (Nacalai, 30264-85), skim milk (BD, 232 100), TRIzol reagent (Life technologies, 15596-018), α-amanitin (Calbiochem, 129741). Antibodies and drugs were stored in small aliquots at RT, 4 or −25°C as recommended by the providers.

### Plasmids

DsRed2-B23 was kindly provided by Dr Angus Lamond (23). SC35-DsRed2 (24), CAG-GFP/RFP (25) were constructed as previously described.

### Equipment for probe synthesis and spectral measurements

DNA was synthesized on a 392 DNA/RNA synthesizer (Applied Biosystems) or a NTS H-6 DNA/RNA synthesizer (Nihon Techno service). Reversed-phase High-performance liquid chromatography (HPLC) was performed on CHEMCOBOND 5-ODS-H columns (10–150 mm) with a Gilson Chromatograph, Model 305, using a UV detector, Model 118, at 260 nm. MALDI-TOF mass spectra were measured with a Bruker Daltonics Reflex. UV and fluorescence spectra were recorded on a UV-2550 spectrophotometer and RF-5300PC spectrofluoro-photometer (Shimadzu), respectively.

### Probe synthesis

NABiT SF 1.0 software was used to predict effective hybridization-sensitive ECHO probes (26). In addition, RNA secondary structures were predicted with CentroidFold (CBRC) and regions of known functions and long stems were avoided. Synthesis of TO dyes was as described (20). Oligonucleotides were synthesized with a DNA autosynthesizer using standard phosphoramidite chemistry (20). The synthesized DNA oligomer containing a diamino-modified nucleotide was cleaved from the controlled pore glass (CPG) support with 28% aqueous ammonia and deprotected according to the protocol for cy5 phosphoramidite. After removal of ammonia from the solution under reduced pressure, the DNA was purified by reversed-phase HPLC, eluted with a solvent mixture of 0.1 M triethylammonium acetate (TEAA) (pH = 7.0) and a linear gradient over 20 min from 5 to 30% acetonitrile at a flow rate of 3.0 ml/min. A solution of the succinimidyl ester of TO dyes (50 equiv to an active amino group of DNA) in N,N-dimethylformamide (DMF) was added to a solution of purified DNA in 100 mM sodium carbonate buffer (pH = 9.0) and incubated at 25°C for 10 min. The reaction mixture was diluted with ethanol. After centrifuging at 4°C for 20 min, the supernatant was removed. The residue was dissolved in a small amount of water and then the solution was passed through a 0.45 μm filter. The product was purified by reversed-phase HPLC on a 5-ODS-H column, eluted with a solvent mixture of 0.1 M TEAA (pH = 7.0) and linear gradient over 28 min from 5 to 40% acetonitrile at a flow rate of 3.0 ml/min). The fluorescent DNA was identified by MALDI-TOF mass spectrometry.

### Cell culture

HeLa cells and MCF7 cells were cultured at 37°C in DMEM supplemented with 10% heat-inactivated FBS, 25 U/ml penicillin and 25 mg/ml streptomycin. SH-SY5Y cells were cultured at 37°C in RPMI 1640 supplemented with 10% heat-inactivated FBS, 25 U/ml penicillin and 25 mg/ml streptomycin. Hippocampus and cerebellum were dissected from P0 and P5 mice respectively and treated with papain. Dissociated neurons were plated in 4-well plate containing 12 mm glass coverslips coated with 0.5 mg/ml poly-L-lysine, maintained in neurobasal-A medium containing B27 supplement and GlutaMAX as previously described (19).

### In-cell spectral imaging

Lambda acquisition mode and meta detector on LSM780 were used to collect and sort emission from a single pixel (marked by the cross in Supplementary Figure S2C) into a series of small wavelength bands. The mean pixel intensity was then plotted against the wavelength, which was compared to the reference spectra to determine the photon source of the fluorescence.

### Kinetic analysis of hybridization-dependent fluorescence activation of the probes

Kinetic measurements were carried out at room temperature using a stopped-flow spectrafluorometer (Model SX20; Applied Photophysics) equipped with dual photomultiplier tubes. Excitation 500 nm, filter set 520/35 nm. About 50 nM probe and 0.35, 0.7, 1.4 or 2.8 μM target oligonucleotides were dissolved separately in phosphate buffered saline (PBS), and mixed by firing the injection piston with simultaneous collection of fluorescence intensity as a function of time with millisecond resolution. All kinetic data represent the average of at least six experiments. Activation time is calculated as the time required for the probe fluorescence to reach half of its maximal intensity. Kinetics of hybridization-dependent fluorescence activation was fitted to the equation, *y* = ce^−*K*^x + A; y is fluorescence intensity; x is lapsed time after mixing, by the software mounted on SX20. Each *K*-value was plotted against the concentrations of target oligonucleotides. Then a linear regression was conducted on the all *K*-values, *K* = *K*_on_[target oligo] + *K*_off_. *K*_D_ was calculated from the equation of *K*_D_ = *K*_off_/*K*_on_.

### Fluorophotospectral measurements

Fluorescence spectra of the probes were measured in DEPC/PBS or in 4XSSC, 0.5 mM EDTA, 10% dextran sulfate, 10% deionized-formamide in Milli-Q water using a cuvette with a 1-cm path length. The excitation wavelength was 514 nm (bandwidths 1.5 nm). Nucleic acids to be hybridized (single stranded DNA, extracted RNA, FBS, etc.) were added directly into the solution containing 0.2–0.5 μM probes in the cuvette and mixed by vortex. Extracted RNA was isolated from chick embryo or adult mouse brain using an RNAqueous Kit (Ambion). An oligotex-dT_30_ mRNA purification kit (TaKaRa) was used to isolate mRNA from total RNA. After mixing, up to 5 min were allowed for hybridization before measurements were taken. To quantify the fluorescence activation of the probes, fluorescence intensities around the emission peak (520–700 nm) were summed and used to calculate the fluorescence intensity ratio of the probes before and after hybridization.

### Transfection of ECHO probes into cultured HeLa cells

ECHO probes were transduced into HeLa cells cultured on 0.17 mm-thickness glass bottom dishes (Matsunami, D111300) at a 0.5 μM final concentration using Lipofectamine2000 reagent. After incubation for 1 h in the probe/lipofectamine/OPTI-MEM solution, the cells were rinsed three times with warmed PBS and observed in culture medium in a live-cell imaging chamber system.

### Microinjection of ECHO probes into cultured HeLa cells

For microinjection, a Femtojet (eppendorf) and Sterile Femtotips II (eppendorf) were used. Probes were dissolved in PBS to a final concentration of 5 μM and microinjected into the cytoplasm of cells.

### ECHO-FISH and immunostaining in fixed cells

Cells were fixed in 4% paraformaldehyde (PFA) for 15 min and permeabilized in 0.1–0.5% tritonX-100/PBS for 5 min on the bench. Cells and tissue were blocked in 1% BSA in DEPC/PBS or 5% skim milk in DEPC/PBS for 30 min before primary antibody incubation (overnight, 4°C). After secondary antibody incubation in the dark for 1 h (fluorescently labeled with Alexa dyes, Invitrogen), 0.2 μM ECHO probes were applied in 4XSSC, 0.5 mM EDTA, 10% dextran sulfate, 10% (for poly (A) RNA) or 25% (for others) deionized-formamide in DEPC-H_2_O and incubated for 3 h to overnight at room temperature. Samples can be imaged immediately after this step or mounted in ProLong mounting medium or CC/Mount™ for later studies.

### SUnSET

To estimate the global translation activity in probe-transfected HeLa cells, 10 μg/ml of puromycin were added to the culture medium. After 30 min of incubation at 37°C, cells were rinsed two times with warm culture medium and incubated in culture medium for 1 h at 37°C. For western-blotting, cells were put on ice and rinsed twice with ice-cold PBS. After two rinses, PEB (protein extraction buffer; 50 mM Tris–HCl (pH 7.4), 150 mM NaCl, 1 mM EDTA, 1% Nonidet, 1% Protease inhibitor cocktail) was added to cells to extract total protein. Protein lysates were mixed with laemmli buffer and boiled for 5 min at 95°C and separated by 10% Sodium dodecyl sulphate-polyacrylamide gel electrophoresis. Proteins were transferred to PVDF membrane by semi-dry or tank blotting. The chemifluorescence from ECL was detected with LAS-4000 imager (Fujifilm). For immunostaining, cells were immediately fixed in 4% PFA and permeabilized in 0.5% tritonX-100/PBS for 5 min on the bench. Cells were blocked in 5% skim milk in PBS for 30 min before primary antibody incubation (overnight, 4°C). After secondary antibody incubation in the dark for 1 h, cells were mounted in ProLong mounting medium for later studies (27).

### RT-qPCR

Total RNA was isolated from probe-transfected HeLa cells with TRIzol (Life Technologies, 15596-018) and reverse transcribed to cDNA using ReverTra Ace qPCR RT Master Mix with gDNA remover (TOYOBO), according to the manufacturer's instructions. Real-time quantitative polymerase chain reaction (PCR) analysis was performed in 10 μl reactions using StepOnePlus™ Real Time PCR system (Applied Biosystems) and SYBR Green PCR Master Mix (Applied Biosystems). Relative gene expression was determined by ΔΔC_T_ method. For PCR amplification, the specific primers, 5′-GTAACCCGTTGAACCCCATT-3′ and 5′-CCATCCAATCGGTAGTAGCG-3′ for 18S rRNA, 5′-GATGTGATTTCTGCCCAGTG-3′ and 5′-ATGACGAGGCATTTGGCTAC-3′ for 28S rRNA, 5′-ACGTGTAGAGCACCGAAAAC-3′ and 5′-ACGATCATCAATGGCTGACG-3′ for U3 snoRNA, 5′-ATCATCAGCAATGCCTCCTGC-3′ and 5′-ATGGCATGGACTGTGGTCATG-3′ for GAPDH were used.

### *In vivo* electroporation of ECHO probes into living mouse cerebellum

*In vivo* electroporation into postnatal mouse cerebella was performed with a previously described protocol to fluorescently label migrating cerebellar cells, with slight modifications (25). Briefly, before electroporation, P7–9 ICR mice were anesthetized by being covered with ice for 6–7 min. The injection microsyringe, MS-NE05 (with 33-gauge needle connected to the anode, ITO Corporation) was stabilized onto the micromanipulator at an angle of 60° (MM-3, Narishige). After three rinses in ethanol and sterile water sequentially. About 0.8 μl of sample (DNA plasmids, Cy5-d(T)_30_ and/or ECHO probes) solutions (in TE with 0.1% fast green dye; injection concentration was 200 ng/μl for ECHO probes and 3 μg/μl for DNA plasmids) were loaded into the injection syringe by pulling back the plunger. The head-neck region of the anesthetized mice was cleaned with 70% ethanol before surgery. The subsequent surgical procedures were carried out on a M80 (Leica) stereomicroscope.

The skin of the head-neck region was cut open along the midline to expose the underlying muscle and skull. The muscle fibers were disconnected with tweezers and a hole was drilled in the skull for injection. The loaded microsyringe was then lowered into the interlobular space between lobule V and VI of the mouse cerebellum in the depth of 0.5 mm. After the sample solution was injected manually, a pair of tweezer-type cathode electrodes (CUY613P3, Nepagene) was hand-held by the experimenter and gently pressed against the sides of the occipital regions. Electrical pulses (6 pulses of 70 V for 50 ms-duration with 150 ms-intervals) were delivered using a pulse generator, CUY21 (Nepagene). The mice were revived at 37°C and returned to the litter until further experiments.

### Imaging target RNA in acute cerebellar slices in P7–9 mice

For *ex vivo* imaging, mouse brains were quickly dissected out and transferred into ice-cold artificial cerebrospinal fluid (ACSF) composed of (in mM): 124 NaCl, 3 KCl, 26 NaHCO_3_, 2 CaCl_2_/2H_2_O, 1 MgSO_4_/7H_2_O, 1.25 KH_2_PO_4_, 10 D-glucose bubbled with a gas mixture of 95% O_2_/5% CO_2_. The brains were embedded in 3% agarose gel and sagittal or coronal slices (300 μm-thickness) were sectioned using a vibratome (linear slicer PRO 7, Dosaka EM). Slices were mounted onto a ϕ27 mm-hole glass-bottom dish (Matsunami, D111400), held by a slice anchor (Warner Instruments, SHD-22CL/15) and soaked in ACSF for live-cell imaging. Single snapshot images and time-lapse images were acquired on a FV1000 inverted laser scanning confocal microscope. In separate experiments, the brains were cut along the saggital midline into halves and ‘collagen-glued’ onto glass bottom dish in an open-book configuration. Individual speckles could be observed in both sectioned slices and also in the halved hemispheres.

### Immunofluorescence analysis and ECHO-FISH on cerebellar slices

For immunofluorescence analysis or FISH on cerebellar slices, sagittal or coronal slices (50 μm-thickness) were sectioned using a vibratome (microslicer DTK-3000W, Dosaka EM) after perfusion fixation through the heart and post-fixation overnight. Then, slices were permeabilized in 0.1% tritonX-100/PBS for 15 min on the bench and blocked in 5% skim-milk in DEPC/PBS for 30 min before primary antibody incubation. After secondary antibody incubation in the dark for 1 h (fluorescently labeled with Alexa dyes, Invitrogen), 0.2 μM ECHO probes were applied in 4XSSC, 0.5 mM EDTA, 10% dextran sulfate, 10% (for poly(A) RNA probe) 25% (for others) deionized-formamide in DEPC-H_2_O and incubated overnight at room temperature. Samples can be imaged immediately after this step or mounted in CC/Mount™ for later studies.

### Drug treatments

HeLa cells were treated with 50 nM of actinomycin D (actD), 100 μM of oxaliplatin (Oxa), 400 μM of cisplatin (Cis) or 50 μM of α-amanitin for 1 h. Acute brain slices were treated with 100 μM of Oxa or 100 nM of actD for 1 h.

### Migration assay

To assess migration of cerebellar granule cells, CAG-GFP and/or D514-U3 solutions were electroporated into P7 ICR mice. After the electroporation, mice were returned to their litter for 3–4 days. Sagittal slices (50 μm-thickness) of P10 or P11 electroporated mice were sectioned using a vibratome (microslicer DTK-3000W, Dosaka EM) after perfusion fixation through the heart and post-fixation overnight. After sectioning, cerebellar slices were stained with DAPI for 5 min and mounted in CC/Mount™ for fluorescence imaging. Migration rates were analyzed with the number of GFP-labeled granule neurons. GFP-labeled granule neurons in the inner granule layers were counted as neurons that have successfully completed migration and GFP-labeled granule neurons in the external granule layers were counted as cells that failed to migrate timely.

### Electroporation and observation of ECHO probes in living chick embryos

*In ovo* or *in dish* electroporation into developing chick embryos was carried out at Hamburger and Hamilton stage (HH stage 9–10 or 8–9 somite stage) or HH stage 22 (Embryonic day 4) as described previously (28–30). To minimize the damage and to limit the range of effects on HH stage 9–10 embryos, a tungsten microelectrode (Negagene, CUY614T) coated with nail polish except for the tip was used as the cathode (31) and a platinum microelectrode (Nepagene, CUY613P1) was used as the anode during electroporation. Probes were injected into neural tubes until overflowing immediately before electroporation. ECHO probes were electroporated at a concentration of 200 ng/μl with charged square pulses of 7 V for 9 ms three times. CAG-RFP expression vector was co-electroporated with probes at 0.5 μg/μl in order to label cells that had been successfully electroporated with the vector DNA together with the probes.

For *in dish* electroporation, HH stage 22 living embryos were removed from the eggs and submerged in PBS immediately before injection of probes. Electroporation and whole mount imaging were done immediately afterward. Heartbeats were monitored to confirm the living state of the embryos during manipulation and imaging. The affected sites indicated by probe fluorescence were dissected and flat-mounted onto normal glass slides for imaging at the single cell level. In a separate set of experiments, we co-electroporated CAG-RFP plasmid vectors to fluorescently label the transduced cells.

To test the long-term efficiency of probes to monitor RNAs in living chick embryos, we electroporated chick embryos at HH stage 9∼10 and followed the fluorescence of probes over 24 h when the embryos reached HH stage 16∼17. The fluorescence of transfected probes and co-transfected CAG-RFP vectors were first imaged *in ovo* within 3 h following electroporation. The embryos were then maintained in the 38°C incubator to allow normal development. About 20–21 h after incubation, the same embryos were removed from the eggs and imaged again.

### Image acquisition, processing and quantification

Large field fluorescence images of electroporated mouse cerebellum were acquired on stereomicroscopy, SZX10 mounted with a microscope digital camera DP80 operated by CellSens (Olympus). Fluorescence images of ECHO probe-microinjected HeLa cells were acquired on Cell Observer mounted with a LED illumination system, Colibri using Plan Apochromat 63× objective operated by AxioVision Rel. 4.8 (Zeiss). Confocal images were acquired either with LSM780 using Plan Apochromat 63× objective operated by ZEN2010 software (Zeiss) or with FV1000 using UPLSAPO 20X, UPLSAPO 60XO and PLAPO 60XO objectives operated by FV10-ASW 4.2 (Olympus). Laser intensities and photomultiplier tube gains were adjusted to achieve nonsaturating fluorescence with minimal bleaching and phototoxicity. Pinhole diameters were adjusted to 1 airy unit for all confocal imaging. Imaging parameters can also be found in figure legends. *In ovo* or *in dish* fluorescence images of living chick embryos were acquired on MZ16 FA (Leica) mounted with DP70 operated by CellSens (Olympus). Specific filter sets were used to separate fluorescence signals: ECHO probes, D514 (ex 500/24–25, dm 520, em 542/25–27); DAPI, (G 365, FT 395, BP 445/50); RFP (ex BP 545/25, dm FT 570, em 605/70).

For time-lapse imaging, cells (in DMEM supplemented with 10% heat-inactivated FBS, 25 U/ml penicillin and 25 mg/ml streptomycin) or acute brain slices (in ACSF) were maintained in live-cell imaging chambers mounted to the microscopes at 37°C supplied with 5% CO_2_ and moist.

Acquired images were processed with the software ZEN 2011 (Zeiss), FV10-ASW 2.1 Viewer (Olympus) and Image J (NIH). Maximum intensity Z-projection was performed to all images. Movement tracking of nuclear foci was performed with the software Imaris (Bitplane) and quantified as diffusion coefficient. To quantify the fluorescence activation of the probes in HeLa cells and granule cells, signal intensity from regions of interest (ROIs) were measured using Image J.

### Statistical analysis

Statistical analysis were done with Excel (Microsoft), Graphpad Prism (Graphpad software) and EZR (32). The *P*-values associated with comparisons in Figure 3 were based on One-way ANOVA and the *P*-values associated with other comparisons were based on Student's *t*-test. Results are plotted as mean ± SEM from three independent experiments, unless otherwise indicated in the figure legends.

## RESULTS

### Design, synthesis and *in vitro* characterizations of ECHO probes

**Figure 1.**
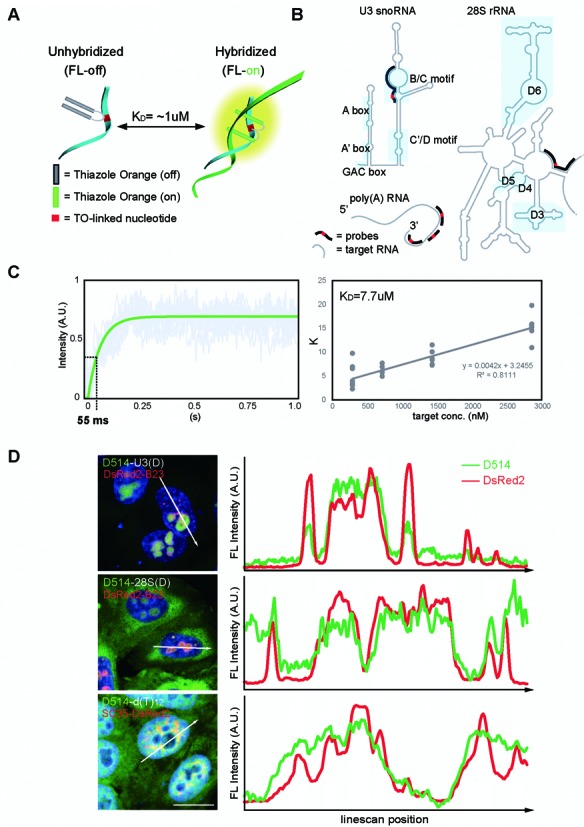
Design, activation kinetics and transcript-specific labeling of ECHO probes. (**A**) Graphic illustration of hybridization-sensitive reversible fluorescence emission of ECHO probes (19). (**B**) Transcript-specific ECHO probes (black lines) generated against U3 snoRNA, 28S rRNA and poly(A) RNA (gray lines). Known functional motifs are labeled along the transcripts (masked areas). (**C**) Kinetics of hybridization-dependent fluorescence activation measured with stopped-flow technique. *Left*: fluorescence intensity of 50 nM probes (y) versus the lapsed time (s) after mixing with 1400 nM target oligonucleotides (x). Gray lines: 10 trials of measurements; green line: fitted curve (*y* = ce^−*K*^x + A). Fluorescence activation occurs within tens of milliseconds in the presence of target oligonucleotides. *Right*,*K*-value plotted against the concentrations of target oligonucleotides. *K* = *K*_on_[target oligo] + *K*_off_, *K*_D_ = *K*_off_/*K*_on_. (**D**) *Left*, Confocal images (LSM780) represent maximum intensity z-projections of HeLa cells overexpressing nuclear foci protein markers (red), probed by transcript-specific ECHO probes (green), and stained with DAPI (blue). *Right*, intensity plots for pixels along the straight lines drawn across individual HeLa cells. Peaks at the same line-scan position represent colocalization of protein markers and target transcripts at nuclear foci (shaded areas). A.U.: arbitrary units; Scale bars: 20 μm. DAPI: 405 nm excitation, 410–500nm detection; D514: 514 nm excitation, 517–552 nm detection; DsRed2: 561 nm excitation, 566–703 nm detection..0.132 μm x 0.132 μm pixel size; 5–50 μs pixel dwell times; 1.5 μm optical slices at 0.75 μm interval up to 3 μm depth.

To target RNA in intranuclear foci such as nucleoli and nuclear speckles, we designed and synthesized ECHO hybridization probes to poly(A) RNA, U3 snoRNA and 28S rRNA (Table 1, Figure 1A and B; (19–20,33). A BLAST search against the Refseq database confirmed single target identities with 100% complementarity. Spectral measurements of the synthesized probes demonstrated hybridization-sensitive, target-specific and concentration-dependent fluorescence activation (Supplementary Figure S1). A kinetic analysis of the fluorescence activation using a stopped-flow technique showed ultrafast activation times of 69.56 ± 1.79, 45.76 ± 1.62 and 47.80 ± 1.58 ms​ and effective affinities of 5.19 ± 2.61, 8.48 ± 4.80 and 8.54 ± 2.25 μM averaged from six independent experiments for three distinct probe-target sets (Figure 1C and Supplementary Table S1). Together the *in vitro* characterization of synthesized probes suggests ultrafast fluorescence detection of RNA targets with high specificity.

**Table 1.**
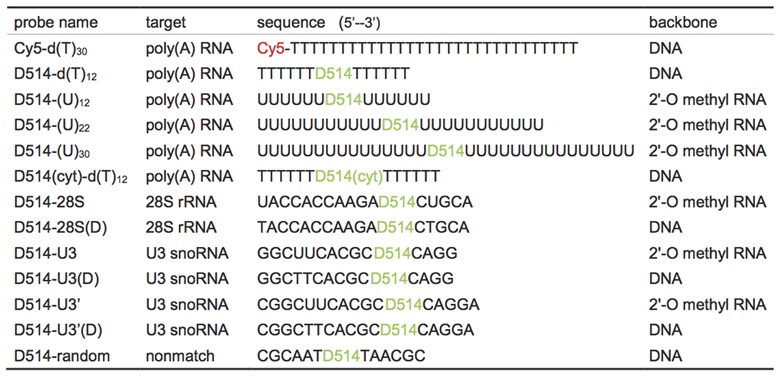
Probes used in this study

We next applied the ECHO probes to *in situ* fluorescence RNA imaging. In D514-d(T)_12_-hybridized HeLa cells, nuclear speckles that contain poly(A) RNA concentrations had over 300 times higher fluorescence intensity when compared to regions outside of cell. This fluorescence ratio was 74-fold higher than that of conventional Cy5-d(T)_12_ probes, owing to the significantly lower background fluorescence of D514-d(T)_12_ (Supplementary Figure S2A). To test target-specificity of detection, U3 snoRNA, 28S rRNA and poly(A) RNA-specific D514 probes were hybridized to HeLa cells exogenously expressing DsRed2-B23 (B23, a nucleolar marker protein) or DsRed2-SC35 (SC35, a nuclear speckle marker protein) and fluorescence colocalization was examined in the fixed nuclei with line-scan plots. Phase-locked fluorescence intensity peaks indicated co-concentrations of the target RNA and nuclear protein markers, thus suggesting labeling specificity for the target RNAs as U3 snoRNA, 28S rRNA, and poly(A) RNA have previously been shown to concentrate at nucleoli and nuclear speckles (Figure 1D and Supplementary Figure S2B).

### D514-(U)_22_ probes reveal stability and immobility of poly(A) RNA foci at nuclear speckles in living HeLa cells

**Figure 2.**
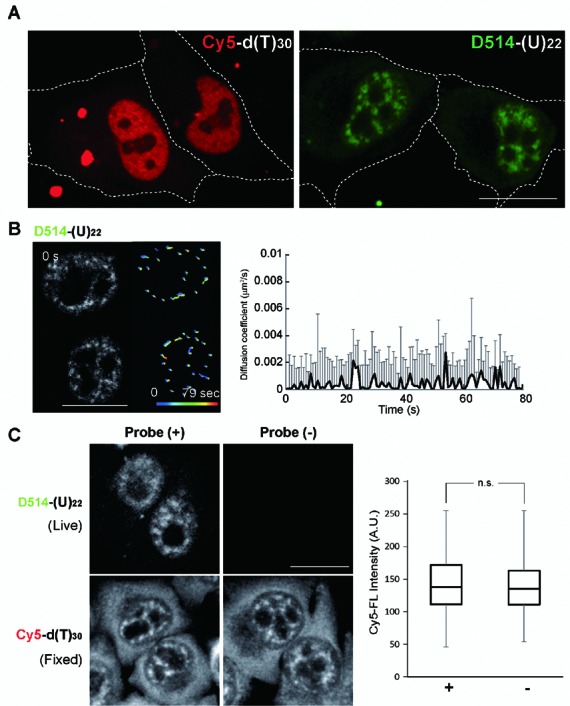
ECHO-liveFISH imaging of poly(A) RNA foci in living HeLa cells reveals immobility. (**A**) Fluorescence images of HeLa cells transfected with Cy5-d(T)_30_ or D514-(U)_22_. Speckled nuclear fluorescence was readily distinguished in the nuclei of D514-(U)_22_ transfected cells but not in the Cy5-d(T)_30_ transfected cells. (**B**) Time-lapse confocal poly(A) RNA images (LSM780) of HeLa cells transfected with D514-(U)_22_ (also see Supplementary Video 1). The track-line presentations monitor the center position of individual speckles over time (progressing from blue to red). Diffusion coefficient of each poly(A) speckle was plotted over time. (**C**) Poly(A) RNA staining in HeLa cells after ECHO-liveFISH imaging. *Top*, D514-(U)_22_ fluorescence in transfected and mock-transfected cells. *Bottom*, after imaging with D514-(U)_22_, cells were fixed, permeabilized and hybridized with Cy5-d(T)_30_ probes followed by Cy5 fluorescence imaging. Quantification of mean Cy5 fluorescence intensity at individual speckles (MFI) indicated comparable endogenous poly(A) concentrations at nuclear foci in D514-(U)_22_-transfected and mock-transfected cells. Scale bars: 20 μm. D514: 514 nm excitation, 517–597 nm detection; Cy5: 633 nm excitation, 638–759 nm detection. 0.264 × 0.264 μm pixel size; 50 μs pixel dwell time.

We next transfected a ribonuclease-resistant poly(A) RNA probe D514-(U)_22_ (U: 2’*O*-methyl-uridine) into living HeLa cells (33,34). Upon transfection, discrete speckles of concentrated fluorescence were observed in the nucleus and weak and diffuse fluorescence was observed in the cytoplasm. Transfection of Cy5-d(T)_30_ however, revealed homogeneous fluorescence distribution in both nucleus and cytoplasm (Figure 2A). An in-cell spectral profile in D514-(U)_22_ transfected cells identified TO as the sole fluorescence source (Supplementary Figure S2C). The lack of fluorescence in the nucleoli is consistent with previous findings that poly(A) RNA is concentrated at ‘interchromatin granule clusters/nuclear speckles’ (1,35). We then performed time-lapse imaging, which revealed stable fluorescence intensity at each individual poly(A) RNA foci over time (Supplementary Figure S2D). The photostability of D514-(U)_22_ allowed us to take a 80-frame movie (continuous imaging for a total of 80 s) of transfected HeLa cells and track the mass center of each individual speckle to quantify mobility. As shown in Figure 2B, diffusion coefficient of individual speckles was 0.0004 ± 0.0021 μm^2^/s throughout the time-lapse imaging, indicating both stability and immobility of poly(A) speckles (Figure 2B and Supplementary Video 1). In order to determine whether the dynamic behavior of endogenous poly(A) RNA was affected by ECHO-liveFISH, we fixed the cells at the end of the experiments and re-probed cells with Cy5-d(T)_30_. Neither fluorescence intensity or cellular distribution of Cy5-d(T)_30_ was affected as compared to the vehicle-transfected cells, indicating that expression level and trafficking of poly(A) RNA were not perturbed (Figure 2C). Poly(A) nuclear speckles labeled by D514-U_22_ were spatially separated from nucleoli where fluorescence of D514-U3 and D514-28S probes concentrated, consistently with the well characterized nuclear locations of these three RNA (Supplementary Figure S2E). Thus, the dynamic imaging of poly(A) RNA in living HeLa cells demonstrates photostability, target-selectivity and usefulness of the probes in monitoring intranuclear RNA foci in living cells.

### Probe labeling and ECHO-liveFISH imaging do not induce metabolic or functional interference to the target RNA

**Figure 3.**
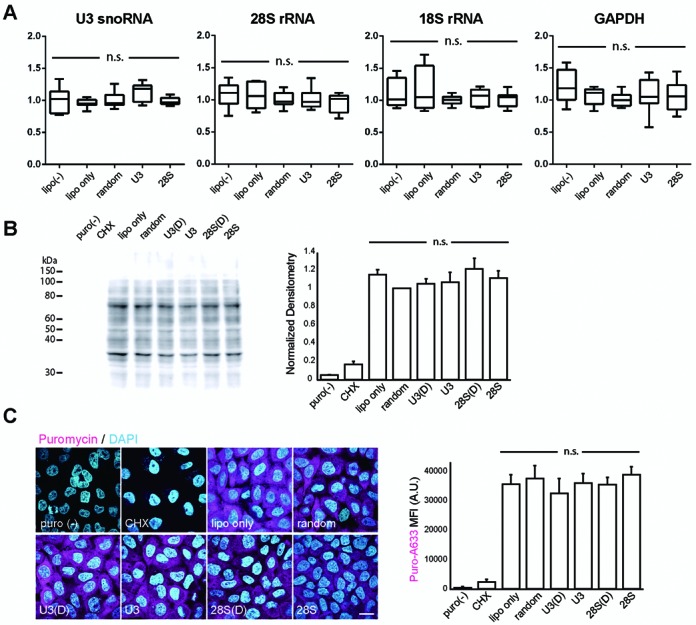
ECHO-liveFISH imaging did not alter the expression level or the function of the target RNA. (**A**) Total RNA was isolated from transfected HeLa cells and specific target RNAs were quantified with RT-qPCR. The graphs show the expression levels of U3 snoRNA, 28S rRNA, 18S rRNA and GAPDH (normalized to ‘D514-random’-transfected cells). Note that no significant difference was induced by transfection of target-specific ECHO probes (one-way ANOVA). (**B**) Estimation of global translational activities in the probe-transfected HeLa cells with puromycin pulse-labeling and immunoblotting (27). Immunodensitometry in each individual lane was analyzed and normalized to that of the ‘D514-random’ lane. *CHX*, cycloheximide, a translational inhibitor. (**C**) *Left*, Confocal images (FV1000) of ECHO probe-transfected HeLa cells immunostained with a puromycin-specific antibody (magenta) and DAPI (cyan). Cellular distribution of newly synthesized proteins was unaffected among cell groups. *Right*, Quantification of mean fluorescence staining intensity (MFI) of puromycin showed undetectable changes in the gross translational activity among cell groups. A.U.: arbitrary units. Scale bar: 20 μm. DAPI: 405 nm excitation, 425–475 nm detection; Alexa 633: 635 nm excitation, 650–750 nm detection; 0.165 × 0.165 μm pixel size; 20 μs pixel dwell times.

To determine whether hybridization-based labeling of ECHO-liveFISH perturbs the expression of the target RNA, we performed RT-qPCR to quantify U3 snoRNA, 28S rRNA, 18S rRNA and GAPDH RNA expression in transfected HeLa cells with >80% transfection efficiency. When compared to cells transfected with a D514 probe with no complementary targets (D514-random), no significant differences were observed among HeLa cells transfected with various probes, indicating that probe interactions did not affect the expression level of the target RNA (Figure 3A). To determine whether the function of the target RNA was affected, we performed SUnSET, a puromycin pulse-chase experiment (puromycin, a structural analog of aminoacyl-tRNA) to estimate the gross translational activity in transfected HeLa cells (Figure 3B, 3C; (27)). Based on the roles of U3 snoRNA in splicing rRNA species, 28S rRNA in ribosomal function and poly(A) tails in regulating translation, we reasoned that altered expression levels and/or function of these RNA targets would result in observable changes in splicing and translation activity. Significant differences in gross translation activity, estimated by immunoblotting (Figure 3B) and immunocytochemistry (Figure 3C), were not observed between the probe- and mock-transfected controls. Taken together these results indicate that (i) the probe labeling of ECHO-liveFISH did not affect the expression level of the target or non-target RNA; (ii) the function of U3 snoRNA to process 18S/28S rRNA splicing remained intact; and (iii) ribosomal function of translation was unaffected. The unaltered RNA expression and function was corroborated with normal cell morphology and proliferation of the transfected HeLa cells.

### *In vivo* labeling reveals target-specificity at RNA intranuclear foci in cerebellar granule cells

**Figure 4.**
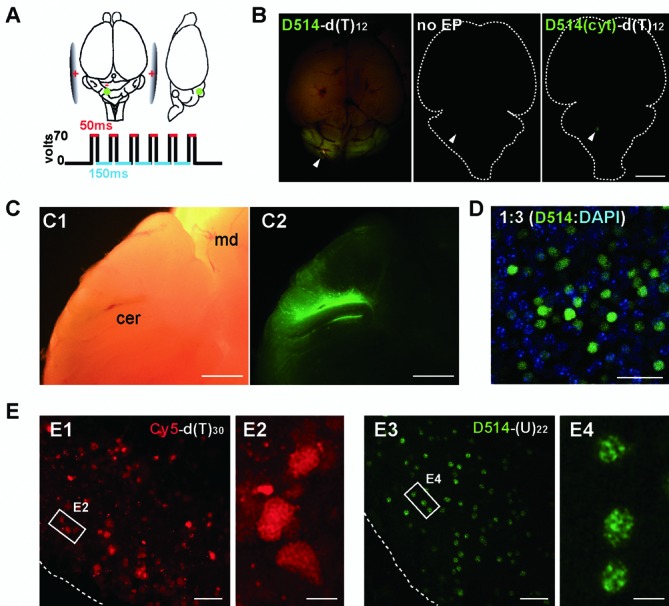
*In vivo* labeling of target RNAs in mouse brains. (**A**) Illustration of the *in vivo* electroporation procedure for delivering ECHO probes into the cerebellum of an anesthetized mouse. Green dots: probe injection sites (500 μm in depth from the surface). (**B**) Dorsal fluorescence view of P7 mice brains electroporated with D514-d(T)_12_ (left), injected with D514-d(T)_12_ with no current delivery (middle), electroporated with a single-mismatched probe that contains cytidine in place of the central thymidine (right). White arrowheads are pointed to the injection sites. (**C**) Bright-field (C1) and fluorescence (C2) views in sagittally partitioned brain halves. cer, cerebellum; md, midbrain. (**D**) Confocal images (LSM780) of permeabilized cerebellar slices stained with DAPI (blue) after *in vivo* electroporation of ECHO probe (green). One out of every three cells in the affected regions contained D514 fluorescence, indicating that delivery efficiency is roughly 30%. DAPI: 405 nm excitation, 410–500 nm detection; D514: 514 nm excitation, 517–597 nm detection; 0.132 × 0.132 μm pixel size; 0.64 μs pixel dwell times. Image stacks of 1.36 μm optical slices at 0.68 μm interval up to 8.9 μm depth. (**E**) Acute cerebellar slices were prepared after *in vivo* electroporation and imaged using a standard confocal laser scanning setup (FV1000). Electroporation of oligonucleotide probes labeled with a conventional dye Cy5-d(T)_30_ showed high fluorescence background at both intracellular and extracellular locations (E1). No distinguishable intranuclear structures were resolved by Cy5-d(T)_30_ labeling (E2). In contrast, D514-(U)_22_ reveals robust fluorescence in nuclei with relatively low background (E3); At higher magnification, D514-(U)_22_ reveals readily distinguishable poly(A) nuclear speckles in individual cerebellar cells (E4). Scale bars: 5 mm (B), 1 mm (C), 20 μm (D, E1, 2), 5 μm (E3, 4). D514: 515 nm excitation, 530–575 nm detection; Cy5: 635 nm excitation, 655–755 nm detection;. 0.207× 0.207 μm pixel size; 8 μs pixel dwell times.

Next we applied ECHO-liveFISH to visualize and monitor the dynamics of RNA intranuclear foci in complex living tissues. To achieve *in vivo* labeling, we modified a DNA electroporation protocol that had been previously used to express fluorescent proteins in migrating cerebellar granule cells (25). Briefly, probes were injected into the space between the cerebellar lobules with a needle anode and a train of six square-pulses of current was delivered between the anode and two cathodes pressed against the sides of the mouse brain (Figure 4A). Following electroporation, the brain was quickly examined under a dissection microscope. Fluorescence signals restricted to the cerebellum was observed, with the highest fluorescence intensity surrounding the injection site (Figure 4B). In the control experiments, we injected D514-d(T)_12_ without delivering current or electroporated D514(cyt)-d(T)_12_, which contained a TO-conjugated cytodine in place of the TO-conjugated thymidine and thus creates a single mismatch between probe and target. In neither experiment did we observe significant fluorescence signal, indicating that intracellular delivery and hybridization to target sequences are required for fluorescence emission (Figure 4B). To observe cells located deeper inside the cerebellum, we partitioned the mouse cerebellum along the sagittal midline or alternatively sectioned 300-μm slices for *ex vivo* imaging at a higher magnification. We observed that a population of granule cells in both the external and inner granule layers had become fluorescently labeled (Figure 4C).

Using DAPI staining following fixation, we estimated the *in vivo* labeling efficiency to be 35.7 ± 9.4%, corresponding to one out of every three granule cells being effectively labeled with D514 probes (Figure 4D). This number is lower than the labeling efficiency in cultured HeLa cells (86.3 ± 4.3%) but higher than that of DNA plasmid expression in cerebellar granule cells (12.0 ± 2.9%, Supplementary Figure S3A). When observed at higher magnification, intracellular fluorescence distribution and individual RNA foci were revealed in cerebellar granule cells. Whereas electroporation of Cy5-d(T)_30_ resulted in noisy background and homogeneous fluorescence in nuclei, readily distinguishable nuclear speckles with concentrated poly(A) fluorescence were observed after electroporation of D514-(U)_22_ (Figure 4E). We compared the use of different types of backbone nucleotides (deoxyribonucleotides vs. 2’-*O*-Me ribonucleotides), probe length (13, 23, and 30nt), and injection concentrations (50 μM vs. 300 μM) and found all conditions revealed speckled patterns, albeit the appearance of focal fluorescence differed (Supplementary Figure S3B). D514-(U)_22_ and D514-(U)_30_ probes revealed the most consistent patterns among individual granule cells at a 50 μM injection concentration (Figure 4E and Supplementary Figure S3B).

### *In vivo* labeling of ECHO probes did not interfere with migration of cerebellar granule cells

**Figure 5.**
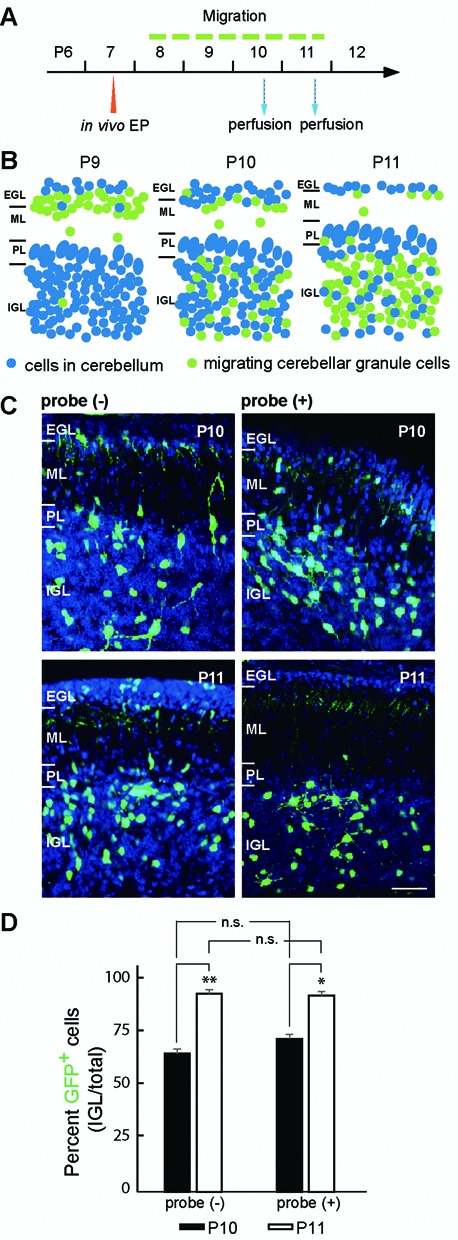
*In vivo* labeling with ECHO probes in cerebellar granule cells did not perturb their migration behavior. (**A**) Experimental scheme to test the potential effect of *in vivo* probe labeling on cerebellar cell migration. CAG-GFP plasmids were electroporated with or without D514-U3 into P7 mouse cerebellum (*in vivo* EP). The animals were then perfused at P10 and P11 with paraformaldehyde and cerebellar cell migration was assessed thereafter. (**B**) Graphic illustration of normal migration process of cerebellar granules cells monitored with *in vivo* GFP electroporation.

To investigate the potentially deleterious effect of *in vivo* RNA labeling on cellular functions, we monitored migration of cerebellar granule cells in cerebellar cortex, a dynamic developmental process of cerebellum between postnatal day 8 and 11. To do so, we co-electroporated RNA-specific ECHO probes with a GFP DNA plasmid into the cerebellar granule cells on postnatal day 7 and monitored GFP expression in the cerebellar layered structures after day 8 (Figure 5A). A successful migration relocates most GFP^+^ granule cells from the external granule cell layer (EGL) into the inner granule cell layer (IGL) by postnatal day 11 (Figure 5B). By calculating the percent of GFP^+^ cells in IGL over total GFP^+^ cells, we quantified migration on P10 and P11 (Figure 5, C and D). Compared to the control cells that were electroporated with only GFP DNA plasmids, cells co-electroporated with GFP plasmids and D514-(U)_22_ completed migration to the similar degree on P10 and P11, indicating that *in vivo* labeling with hybridizing ECHO probes did not perturb the migration behavior of the cerebellar granule cells.

### Specific focal localizations of U3 snoRNA, 28S rRNA and poly(A) RNA verify *in vivo* labeling specificity

**Figure 6.**
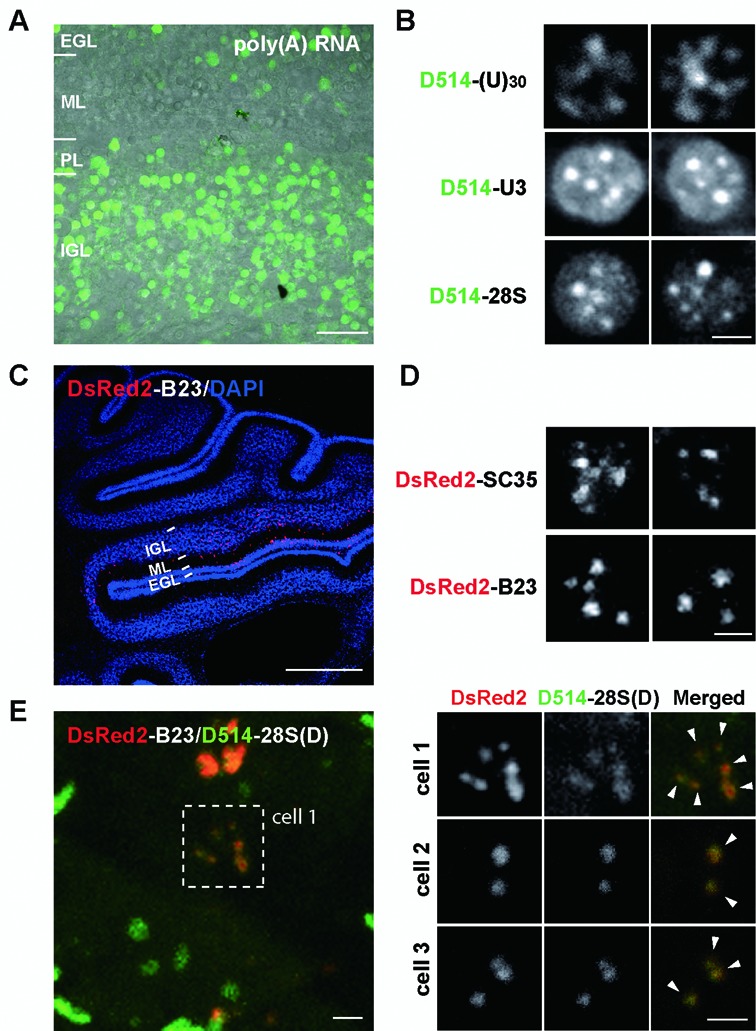
ECHO-liveFISH imaging of target RNA intranuclear foci in acute mouse brain tissues. (**A**) A representative confocal image from acute cerebellar slices prepared soon after *in vivo* electroporation. (**B**) Confocal images (FV1000) of poly(A), U3 snoRNA and 28S rRNA in individual cerebellar granule cells after electroporation. Intranuclear foci containing target RNA concentrations are readily distinguished. The number and shape are consistent with foci of nuclear speckles and nucleoli. (**C**) A confocal image of a DsRed2-B23 electroporated mouse cerebellum stained with a DsRed2-specific antibody (red) and DAPI (blue). (**D**) High magnification confocal images of DsRed2-SC35 and DsRed2-B23 in individual nuclei. Note the irregular shapes of DsRed2-SC35 speckles and round-shaped DsRed2-B23 foci. (**E**) Colocalization between DsRed2-B23 and D514-28S at the nucleoli of electroporated granule neurons. P10 mice expressing DsRed2-B23 DNA plasmids were perfused and cerebellar slices were processed for DsRed2 and DAPI staining simultaneously with D514-28S hybridization. Overlapping DsRed2 and D514 fluorescence at nuclear foci indicates colocalization between B23 proteins and 28S rRNA (white arrowheads). EGL: external granule layer, ML: molecular layer, PL: Purkinje layer, IGL: inner granule layer. Scale bars: 20 μm (A), 2.5 μm (B, C2, D) and 1 mm (C1). DAPI: 405 nm excitation, 425–475 nm detection; D514: 515 nm excitation, 530–575 nm detection; DsRed2: 561 nm excitation, 566–703 nm detection. 0.207 × 0.207 μm pixel size; 8 μs pixel dwell times.

How faithfully does the localized probe fluorescence reflect intranuclear target RNA? It is possible that the *in vivo* electroporation delivery procedure and the far more complex cellular environment of the living tissues could bias the probe localization and behaviors. A large number of fluorescently labeled cerebellar cells allowed us to use the well characterized localization patterns of poly(A), U3 snoRNA and 28S rRNA to verify *in vivo* labeling specificity (Figure 6A). After *in vivo* electroporation, distinct nuclear patterns of fluorescence were observed, which were indistinguishable from those in the cultured cerebellar cells (Figure 6B and Supplementary Figure S3C). To further confirm labeling specificity, we overexpressed DsRed2-B23 or DsRed2-SC35 in living mouse cerebella. Cerebellar granule cells expressing DsRed2 fluorescence were detected in the EGL and IGL layers of cerebellar lobule V and VI adjacent to the injection site (Figure 6C). The U3 snoRNA and 28S rRNA foci largely resembled those labeled with DsRed2-B23 and the poly(A) speckles resembled those labeled with DsRed2-SC35 (Figure 6D). We further hybridized D514-28S(D) and D514-d(T)_12_ to brain slices expressing DsRed2-B23 or SC35-DsRed2. Colocalization between DsRed2 and the RNA probe fluorescence at intranuclear foci confirmed detection specificity *in vivo* (Figure 6E and Supplementary Figure S3, D and E). The molecular specificity of labeling was further confirmed for 28S rRNA and U3 snoRNA probes as both probes were targeted to nucleoli. Fluorescence emission of 28S rRNA and U3 snoRNA probes was strictly activated by target RNA sequences, showing no evidence of cross-activation (Supplementary Figure S4). The *in vivo* specific detection of RNA at intranuclear foci with ECHO probes is attributed to the reduced noise level and target-dependent fluorescence emission because a single mismatch or random sequence in the ECHO probes diminished the fluorescence (Supplementary Figure S5).

### Differed dynamic regulation of RNA foci between cultured HeLa cells and cerebellar granule cells within living brain tissue

**Figure 7.**
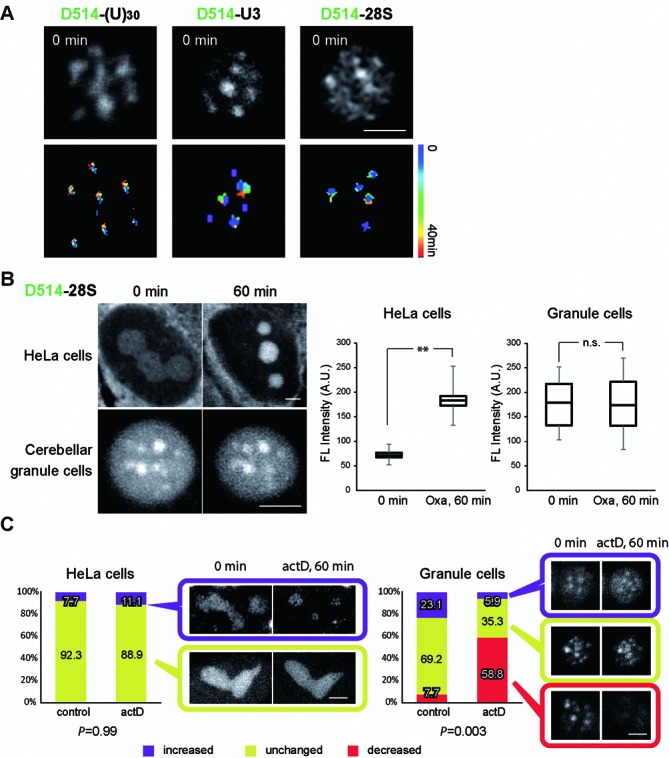
ECHO-liveFISH reveals differential dynamic regulation of RNA foci in ‘*in vivo*’ and ‘*in vitro*’ cells. (**A**) Time-lapse confocal imaging (FV1000) of poly(A), U3 snoRNA and 28S rRNA foci in cerebellar granule cells. *Top*, a snapshot of nuclear foci imaged in acute cerebellar slices after *in vivo* electroporation. *Bottom*, Track-line presentations of individual fluorescent foci over time (progressing from blue to red). (**B**) HeLa cells and cerebellar granules cells were treated with Oxaliplatin (Oxa, 100 μM) for 1 h and 28S rRNA nuclear foci were imaged with D514-28S. *Left*, Confocal images of D514-28S foci in HeLa cells and in cerebellar granule cells before and after Oxa treatment. Condensed 28S rRNA foci fluorescence upon Oxa treatment was observed in HeLa cells but not in cerebellar granule cells. *Right*, Quantification of mean D514 fluorescence intensity at individual foci (MFI) in HeLa cells and granule cells. Fluorescence intensity increased by two folds in HeLa cells after 1 h Oxa treatment, whereas that in granule cells it was unaffected. **P < 0.001. n.s.: not significant. (**C**) Population distributions of HeLa cells and cerebellar cells with increased (purple), unchanged (chartreuse) or decreased (red) numbers of 28S rRNA foci in response to 1 h actinomycin D treatment (actD, 100 nM; transcriptional inhibitor). Representative confocal images of each cell population before and after treatment are shown in pairs. Cerebellar granule cells showed higher response heterogeneity than HeLa cells. A significantly increased portion of cerebellar cells showed decreased number of 28S rRNA foci to less than half, indicating dissipation. Scale bars: 2.5 μm. D514: 515 nm excitation, 500–600 nm detection; 0.207 × 0.207 μm pixel size; 8 μs pixel dwell times.

The photostability of the probes and the high resolution of the confocal fluorescence microscope setup allowed us to perform time-lapse imaging to monitor individual nuclear foci containing concentrations of poly(A), U3 snoRNA and 28S rRNA labeled *in vivo*. We imaged living brain slices in a 37°C humidified chamber (95% O_2_/5% CO_2_) mounted on a FV1000 confocal microscope and tracked the movement of individual foci by monitoring their position and fluorescence intensity over time. Track-line presentations suggest that like poly(A), U3 snoRNA and 28S rRNA nuclear structures are stable with little movement in the living cerebellar granule cells, (Figure 7A and Supplementary Videos 2, 3 and 4). Since ECHO-liveFISH can label nuclear RNA in both cell lines and in living animals, we asked whether the dynamic regulation of the nuclear foci is similar between cerebellar granule cells in complex tissue and cultured HeLa cells. A panel of drugs that had been previously shown to affect nucleoli function (actinomycn D (actD), oxaliplatin (Oxa), cisplatin and α-amanitin; (36) were screened for their effect on 28S rRNA foci in HeLa cells. Dramatic changes in the foci fluorescence intensity and/or morphology were found in HeLa cells within one hour after Oxa (100 μM) and actD (50 nM) treatments (Figure 7B, C and Supplementary Figure S6). We then challenged cerebellar granule cells in acutely prepared brain slices with Oxa and actD. After 1-h treatment with Oxa (100 μM), we could not detect any morphological changes in the 28S rRNA foci (Figure 7B). Quantification of mean fluorescence intensity at individual foci demonstrated significant condensation of 28S rRNA in cultured HeLa cells upon Oxa treatment whereas 28S rRNA foci in cerebellar tissue remained the same as in vehicle-treated samples (Figure 7B).

We then challenged HeLa cells with actD treatment (100 nM). In contrast to Oxa, actD treatment decreased 28S rRNA fluorescence in nucleoli and nucleoplasm. Although the majority of HeLa cells responded to actD treatment with unchanged numbers of 28S rRNA foci in the nuclei (88.9% HeLa cells), the rest of the cells responded with increased numbers of 28S rRNA foci both in vehicle-treated and actD-treated cells, indicating that it was not a actD-specific response (Figure 7C). When cerebellar granule cells in brain slices were challenged with actD, a significant portion of cells (58.8%) responded by decreasing the numbers of foci to less than half, suggesting that actD induced dissipation of 28S rRNA foci in these cells. A significant difference between the vehicle- and actD-treated samples was observed in cerebellar granule cells (*P* = 0.003), but not in HeLa cells (*P* = 0.99). These results suggest that 28S rRNA foci responded to actD treatment with a bigger heterogeneity in cerebellar granule cells than in HeLa cells; actD induced dissipation of 28S rRNA foci in cerebellar granule cells but not in HeLa cells (Figure 7C). Taken together, 28S rRNA foci labeled in living cerebellar granule cells through *in vivo* electroporation responded to Oxa and actD in a fundamentally different manner from cultured HeLa cells. Is this difference restricted to HeLa cells? We further tested the effect of Oxa and actD in MCF7 (breast cancer cells), SH-SY5Y (neuroblastoma), primary cultures of hippocampal neurons and primary cultures of cerebellar granule cells (Supplementary Figure S7). We found condensation of 28S rRNA foci after Oxa treatment in MCF7, SH-SY5Y, cultured hippocampal neurons, but not in cultured cerebellar granule neurons. Dissipation of 28S rRNA foci after actD treatment was observed in cultured cerebellar granule cells, but not in other tested cells (Supplementary Figure S7). Thus the dynamic response of 28S rRNA foci to Oxa and actD was consistent between cultured cerebellar granule cells cells (DIV6) and acutely prepared cerebellar slices, but was different in other tested cells including cultured hippocampal neurons.

### Specific labeling without detectable cytotoxicity in developing chick embryos

**Figure 8.**
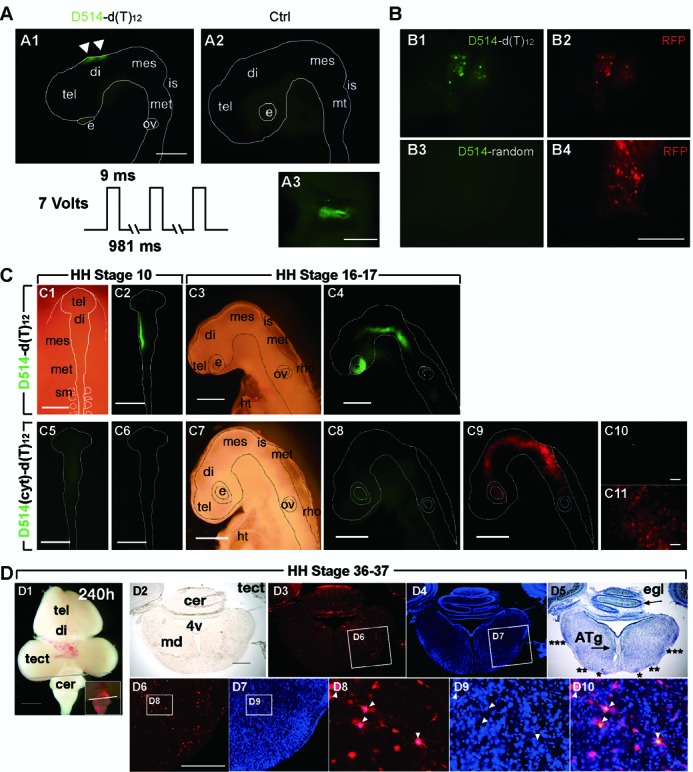
*In vivo* imaging of poly(A) RNA in living chick embryos. (**A**) Micrographs of the head region of a developing chick embryo after *in ovo* electroporation of D514-d(T)_12_. A1: fluorescence observed in the diencephalon region between the electrodes following electroporation; A2: low fluorescence background was observed when D514-d(T)_12_ was injected into the neural tube but no current pulses were applied; A3: dorsal view of the fluorescent region after the chick brain was dissected into phosphate saline buffer (PBS). (**B**) CAG-RFP was co-electroporated either with D514-random or with D514-d(T)_12_ into the diencephalon region. After 4 h to allow fluorescent protein expression, red fluorescence expressing cells also contained D514-d(T)_12_ activity, while almost no D514-random fluorescence was observed regardless of RFP expression. (**C**) Top row, bright field (C1, 3) and fluorescent views (C2, 4) of a developing chick embryo electroporated with D514-d(T)_12_ at HH stage 10, observed at HH stages 10 (C1, 2) and 16–17 (24 h later than HH stage 10; C3, 4). Bottom row, bright field (C7) and fluorescent views (C5, 6, 8–11) of a developing chick embryo electroporated with D514(cyt)-d(T)_12_ and CAG-RFP DNA plasmids at HH stage 10. C6, C8: D514 fluorescence; C6, C9, C11: RFP fluorescence. (**D**) Bright-field and fluorescent views of a developing chick embryo at HH stage 36–37, 240 h after electroporation. D1: ventral view of the chick embryo; the line indicates the position of the cryosection slice viewed in D2–10. D2: bright field; D3: RFP fluorescence (red); D4: DAPI (blue); D5: nissl staining (blue); D6–9: magnified images of the boxed areas in D3, D4; D10: an overlaid image of D8 and D9. tel: telenchephalon, di: diencephalon, mes: mesencephalon, is: isthmus, met: metencephalon, rho: rhombencephalon, e: eye, ov: otic vesicle, sm: somite, ht: heart. Scale bars: 500 μm (A1, B4, C1–9), 100 μm (A3, C10, 11, D6).

To test whether the ECHO-liveFISH method can be generalized to other model organisms we electroporated poly(A) RNA probe D514-d(T)_12_ into the diencephalon region of developing chick embryos using a modified *in ovo* electroporation technique (30). Fluorescence was immediately observed in the diencephalon surrounding the electroporation site (Figure 8A). To confirm labeling specificity, we co-electroporated plasmid DNA cag-RFP with D514-d(T)_12_ or D514-random. RFP fluorescence was observed 4 h after electroporation and the fluorescence of D514-random was not detected concurrently in RFP-positive cells, confirming our previous results in the mouse brain that intracellular delivery and specific hybridization are essential for D514-d(T)_12_ fluorescence (Figure 8B).

To determine whether the RNA labeling can be maintained over multiple developmental stages, we electroporated D514-d(T)_12_ in one side of the diencephalon and mesencephalon regions of a chick embryo at HH stage 10 and continuously monitored the fluorescence up to HH stage 16–17 over 24 h (Figure 8C; (29). Substantial D514-d(T)_12_ fluorescence was retained in the eye and the rhonbencephalon regions derived from the initial diencephalon and mesencephalon regions at HH stage 10, where the cells containing active probes had successfully migrated to. The embryos retaining D514-d(T)_12_ activity at HH 16–17 demonstrated normal morphology, indicating that the sustained probe activity did not interrupt the normal developmental process. In control experiments, the single mismatch probe D514(cyt)-d(T)_12_ showed no significant fluorescence, whereas RFP expression indicated a successful electroporation (Figure 8C).

We continued to monitor the embryos up to 240 h after the electroporation of D514-d(T)_12_. Probe fluorescence was no longer detectable at 240 h (HH stage 36–37; Figure 8D); however, RFP fluorescence-containing cells were widely distributed in the embryo. DAPI and Nissl staining revealed normal gross anatomy, and extended neurites were observed; thus, probe activity did not interfere with either cell migration or cell differentiation (Figure 8D). Collectively, no cytotoxicity after electroporation of D514-d(T)_12_ and RFP plasmid DNA was detected in the developing chick embryos during time-lapse imaging.

## DISCUSSION

We have devised a novel live-cell RNA imaging method, ECHO-liveFISH, by which unmodified, native RNA concentrations at intranuclear foci can be fluorescently labeled both *in vitro* and *in vivo* with simple, highly reproducible and effective nucleic acids delivery techniques such as lipofection, *in vivo* electroporation and microinjection. Whereas lipofection and microinjection have been mainly applied to cultured cells, *in vivo* electroporation has been applied to living model organisms such as mice, zebrafish, *Xenopus, Drosophila* and chicks with tissues- and organs-specificity. After probe delivery, co-labeling with protein markers confirmed the molecular recognition specificity, while the high s/n ratio and fast detection kinetics allowed for real-time visualization of intranuclear RNA foci dynamics. The labeled foci of poly(A), U3 snoRNA and 28S rRNA were largely stable and immobile under normal conditions, but could undergo dynamic changes upon stimulation. Chick embryos containing the D514-d(T)_12_ probe activities successfully followed their developmental course over multiple stages (HH10–37), suggesting the potential use of this technique to study stage-dependent dynamic changes in gene expression in a continuously growing chick embryonic nervous system.

Compared to other currently available techniques, ECHO-liveFISH benefits from being able to potentially target any given RNA in an organism without genetic engineering. Moreover, ECHO-liveFISH is, to our knowledge, the only fluorescent imaging method that can be used to label and visualize RNA concentrations in the nucleus of living multicellular organisms. Although real-time tracking of endogenous poly(A) RNA movement in the nucleus has been performed by using oligo d(T) labeled probes with caged and uncaged fluorescein in myoblasts (37–39), or by using 2’-*O*-methyl-(U) labeled probes by TAMRA or Alexa fluorophores in various cell lines (40,41), these studies have been restricted to immortalized cell lines and never been applied to native tissues or living animals. Further advantages offered by ECHO-liveFISH for *in vivo* RNA imaging are simple and effective delivery, photostability and brightness, reversible fluorescence emission, and data acquisition with standard confocal imaging systems. ECHO probes can be synthesized using standard organic synthesis equipment and an automated DNA synthesizer. Alternatively, ECHO probes can be purchased from Danaform under the name of Eprobe or Eprimer.

An important observation made in this study is the fundamental differences in RNA foci dynamics between the native cells in complex tissue and immortalized HeLa cells. The condensation of 28S rRNA at nucleoli upon oxaliplatin treatment in cancer cell lines and cultured hippocampal neurons was not observed in cerebellar granule cells cultured *in vitro* or acutely prepared from *in vivo*. Given that oxaliplatin was applied at effective concentrations as a chemotherapeutic drug, the insensitivity of 28S rRNA foci in cerebellar granule cells is confirmatory of the *in vivo* effect of the drug. Conversely, the actinomycinD-induced dissipation of 28S rRNA foci in cerebellar granule cells was observed in cultured cerebellar granule cells but not in other tested cells including cultured hippocampal neurons, suggesting independent regulatory mechanisms. Cellular context-dependent foci biogenesis of expanded RNA repeats has been reported for *DMPK* transcripts in myotonic dystrophy type 1, *FMR1* transcript in fragile X retardation syndrome, *JPH3* in Huntington's disease-like 2 (42). Although systematic studies have to be carried out in order to conclude the differences and to explain whether the difference is due to the mitotic states of the cells, metabolic signaling pathways, or other alternative mechanisms, our observation demonstrates the importance of suitable *in vivo* RNA imaging methods to address the nature of nuclear RNA foci dynamics in their endogenous context. Another important observation made in this study is that the labeling with hybridization-sensitive fluorescent deoxyribonucleotide probes or chemically modified 2’-*O*-methyl-ribonucleotide probes labeled with TO dyes do not appear to affect the expression, processing, trafficking and/or function of the target RNA. It is possible that the nuclear accumulation of the probes, the relative quick dissociation rates (up to hundreds of milliseconds), the toleration of 2’-*O*-methyl-ribonucleotide by RNase enzymes, alone or in combination underlie this observation.

The major limitation of ECHO-liveFISH in its current form is the lack of amplification mechanisms of the fluorescence signal, which is critical for detecting transcripts of lower abundance. The current method relies on focal concentrations of RNA for effective detection and specificity validation. To increase detection sensitivity, tandem repeats of hybridization sites have been used to tag target RNA, at the expense of losing the ability to directly study native RNA (24). Chemical optimizations have been made to increase sensitivity, to achieve multi-color imaging (43,44) and to shift to near-infrared emission (45,46). However, the usefulness of ECHO probes in imaging native RNA in living complex tissues has yet to be tested. With recently developed nuclease-resistant TO-labeled DNA forced intercalation oligonucleotide probes (FIT), native oskar mRNPs in living *Drosophila melanogaster* oocytes can be tracked with as few as two probes (47,48). Yet challenges remain in detecting less abundantly expressed genes and in tracking their movement to shed light on RNA transport, localization and functional interactions in complex tissue. Advanced imaging techniques may also be exploited for detection with a higher s/n ratio. Furthermore, the herein method is not suitable for intravital imaging of gene expression (49) but rather restricted to understanding the fundamental cell biology of RNA foci dynamics in living cells and complex tissue.

Many discrete and punctate nuclear bodies such as histone locus bodies, nuclear gems and Cajal bodies, PML bodies, paraspeckles, nucleoli, nuclear speckles containing coding and noncoding RNA have been discovered but their functions are not wholly understood (50). An increasing number of intranuclear foci containing long non-coding RNAs such as *NEAT1, Malat1, Gomafu* and Men ϵ/β are also identified but their biogenesis pathways and functions remain poorly understood. Furthermore, RNA foci with concentrations of expanded RNA repeats such as CUG, CCUG, CGG, CAG, AUUCU are identified as characteristic molecular hallmarks of pathogenesis in human diseases such as myotonic dystrophy, fragile X- associated tremor ataxia syndrome, Huntington's disease-like 2 and spinocerebellar ataxia, but it is not clear whether RNA foci are causing the pathology or accompanying the symptoms in these neurodegenerative diseases (42). The current state of intranuclear RNA detection method does not allow functional tests of the RNA concentrations involved in these assemblies. The potential use of ECHO-liveFISH in studying the dynamic distribution of the many functionally unknown intranuclear RNA foci in primary tissues to other structural and functional entities in transcription, genome dynamic regulation, RNA splicing and processing, signalling, may lead to elucidating the biogenesis and functions of these macromolecular complexes. In our opinion, a method such as ECHO-liveFISH RNA imaging for determining the behavior of RNA foci organization *in vivo* offers complementary benefits to other *in vivo* RNA imaging techniques, such as molecular beacons (51,52), the MS2-GFP system (53,54) and the split-GFP/pumilio RNA binding system (55,56). Intravital RNA imaging in a variety of eukaryotic organisms is also desirable. Such technologies mark the beginning of *in vivo* RNA imaging era that will allow the measurement of quantity, location and timing of gene expression in both healthy and diseased living organisms, thus revealing a more comprehensive picture of gene expression in living organisms.

## SUPPLEMENTARY DATA

Supplementary Data are available at NAR Online.

SUPPLEMENTARY DATA
