# Supplementary material for: ECHO-liveFISH: in vivo RNA labeling reveals dynamic regulation of nuclear RNA foci in living tissues
Source: Nucleic Acids Res. 2015 Jun 22;43(19):e126. doi: 10.1093/nar/gkv614 (PMC4627062; doi:10.1093/nar/gkv614)
Supplement: SUPPLEMENTARY DATA [file supp_43_19_e126__index.html]

ECHO-liveFISH: in vivo RNA labeling reveals dynamic regulation of nuclear RNA foci in living tissues — SUPPLEMENTARY DATA 

# ECHO-liveFISH: *in vivo* RNA labeling reveals dynamic regulation of nuclear RNA foci in living tissues

## SUPPLEMENTARY DATA

- SUPPLEMENTARY DATA
- SUPPLEMENTARY DATA
- SUPPLEMENTARY DATA
- SUPPLEMENTARY DATA
- SUPPLEMENTARY DATA
