# Supplementary material for: ECHO-liveFISH: in vivo RNA labeling reveals dynamic regulation of nuclear RNA foci in living tissues
Source: Nucleic Acids Res. 2015 Jun 22;43(19):e126. doi: 10.1093/nar/gkv614 (PMC4627062; doi:10.1093/nar/gkv614)

Supplementary information for

ECHO-liveFISH: *in vivo* RNA Labeling Reveals Dynamic Regulation of Nuclear RNA Foci in Living Tissues

Ikumi Oomoto[1,2], Asuka Suzuki-Hirano[3,4], Hiroki Umeshima[1], Yong-Woon Han[1], Hiroyuki Yanagisawa[5], Peter Carlton[1], Yoshie Harada[1,2], Mineko Kengaku[1,2], Akimitsu Okamoto[5,6], Tomomi Shimogori[3], Dan Ohtan Wang[1,*]

[1] Institute for Integrated Cell-Material Sciences (WPI-iCeMS), Kyoto University, Yoshida-Honmachi, Sakyo-ku, Kyoto, 6068501, Japan
[2] Graduate School of Biostudies, Kyoto University, Yoshida-Honmachi, Sakyo-ku, Kyoto, 6068501, Japan
[3] Brain Science Institute, RIKEN, Hirosawa, Wako City, Saitama, 3510198, Japan
[4] JSPS Research fellow
[5] Advanced Science Institute, RIKEN, Hirosawa, Wako City, Saitama, 351-0198, Japan
[6] Research Center for Advanced Science and Technology, University of Tokyo, Komaba, Meguro-ku, Tokyo, 153-8904, Japan

* To whom correspondence should be addressed. Tel: +81-75-753-9851; Fax: +81-75-753-9820; Email: dwang@icems.kyoto-u.ac.jp

SUPPLEMENTARY TABLE AND FIGURES LEGENDS

Supplementary figure 1. **Sequence-selectivity and concentration-dependent fluorescence activation of ECHO probes.** (A) Spectral measurement of 0.2 μM D514-$(U)_{12}$ in vehicle solution (4XSSC, 0.5 mM EDTA, 10% dextran sulfate, 10% formamide), in the presence of poly(A)-depleted fraction of RNA (orange), or in the presence of poly(A)-enriched fraction of RNA (green) extracted from adult mouse brain (5 ng/μl), or vehicle solution alone (aqua). (B) Fluorescence on/off ratio of D514-$(U)_{12}$ (0.5 μM) in response to FBS (4 μg/μl), yeast tRNA (1 μg/μl), total RNA extracted from mouse brain (20 ng/μl), poly(A)-depleted total RNA (20 ng/μl), poly(A)-enriched RNA (4 ng/μl), polyadenylic acids (10 μM base concentration), and oligo d$(A)_{13}$ (1μM). *F*on/off: fluorescence intensity (520-700 nm) of hybridized D514-$(U)_{12}$ (0.5 μM) divided by the fluorescence intensity (520-700 nm) of unhybridized D514-$(U)_{12}$ (0.5 μM). *F*on/off of 1.0 indicates no fluorescence activation. (C) Fluorescence on/off ratio of D514-$(U)_{22}$ (*Y*-axis) is plotted against the logarithmic concentrations of hybridizing oligonucleotide d$(A)_{23}$ (*X*-axis) and shows a linear relationship between 10^{-7} and 10^{-5} M of d$(A)_{23}$. (D) Spectral measurement (535-700 nm) of poly(A), U3 snoRNA, and 28S rRNA probes (0.2 μM) in the absence (gray) or presence (green) of complimentary DNA oligonucleotide solutions (0.2 μM). D514-random was mixed with d$(A)_{23}$ where no fluorescence activation was observed (on/off ratio of 1.1).

Supplementary table 1. A summary of the effective binding affinities of the synthesized ECHO probes to their target oligonucleotides and RNA binding affinities in a variety of previously described technologies.

Supplementary figure 2. **ECHO probes detect specific transcripts in both permeabilized and living HeLa cells.** (A) Confocal images of permeabilized HeLa cells probed by Cy5-d$(T)_{13}$ and D514-d$(T)_{12}$ (1 μM). The average intensity in the red box represents the detection signals (F_{sig}), whereas the average intensity in the blue box represents background fluorescence (F_{bg}). (B) Confocal images of permeabilized HeLa cells expressing protein markers DsRed2-B23 and SC35-DsRed2 and probed with transcript-specific ECHO probes. (C) In-cell spectral measurement of D514-$(U)_{22}$ transfected HeLa cells to identify the source of fluorescence to be thiazole orange. Fluorescence intensity was plotted against the emission wavelength. The spectral profiles of EGFP, D514, and DsRed2 clearly distinct from each other which can be used to identify the fluorophores. (D) Fluorescence intensity (*Y*-axis) at individual nuclear speckles in living D514-$(U)_{22}$-transfected HeLa cells is plotted over time. Initial fluorescence intensity was set to 100%. (E) Epifluorescence images of D514-U3, D514-28S, and D514-$(U)_{22}$ probes microinjected into living HeLa cells. Black arrowheads point to nucleoli where D514-U3 and D514-28S fluorescence were concentrated, but not poly(A). Scale bar: 20 μm.

Supplementary figure 3. **Detection of subnuclear RNA concentrations in acute slices and permeabilized cerebellar granule neurons in primary cultures.** (A) Estimated delivery efficiency of ECHO probe into HeLa cells with lipofection (cultured cells), *in vivo* electroporation to mice cerebella (probe EP), and expression of DNA plasmids after *in vivo* electroporation to mice cerebellar (plasmid

EP) was quantified as percent of fluorescent cells over total cell population (counted by DAPI staining). (B) Confocal images of cerebellar granule cells after electroporation to test the optimal usages of probes. We varied backbone nucleotide components (deoxynucleotide vs. 2'-*O*-Methyl ribonucleotides), probe length (13, 23, 31 nt) and injection concentrations (50 μM and 300 μM). Under all conditions, fluorescent intranuclear foci were observed albeit more distinguishable patterns were observed when longer 2'-O-Methyl ribonucleotide probes were used. (C) Confocal images of permeabilized primarily cultured cerebellar granule cells hybridized with D514-U3(D), D514-28S(D) and D514-d$(T)_{12}$. The nuclear foci largely resembled those observed in acutely prepared living brain slices. (D) Confocal images of mouse cerebellar cells electroporated with a SC35-DsRed2 DNA plasmid. Three days after electroporation, mice brains were fixed, sliced, and immunostainied with a DsRed2 antibody. (E) Colocalization between SC35-DsRed2 and D514-d$(T)_{12}$ at nuclear speckles of electroporated granule cells. Scale bar: 5 μm (B, D, E), 10 μm (C).

Supplementary figure 4. **Sequence-specific fluorescence emission of 28S rRNA and U3 snoRNA probes.** D514-28S(D) and D514-U3(D) probes (0.2 μM) were mixed with either a complementary DNA oligonucleotide in 28S rRNA or in U3 snoRNA (0.2 μM) and incubated in 4XSSC, 0.5 mM EDTA, 10% dextran sulfate, 10% formamide for 5 minutes at RT. Fluorescence emission was then measured with spectrofluorophotometry (535-700 nm). Gray lines, probes alone; green lines, 28S rRNA probe-target pair or U3 snoRNA probe-target pair; orange lines, U3 snoRNA probe-28S rRNA pair or 28S rRNA probe-U3 snoRNA pair.

Supplementary figure 5. ***In vivo* labeling with ECHO probes in living mouse brains suggests specific target-dependent fluorescence with high s/n ratio.** *In vivo* electroporation of D514-d$(T)_{12}$ detected concentrated fluorescence in nuclei of cerebellar granule cells. In contrast, electroporation of Cy5-d$(T)_{30}$ reveals fluorescence background both intracellularly and extracellularly without revealing any distinguishable nuclear structures associated with nuclear speckles. Electroporation of D514(cyt)-d$(T)_{12}$ and D514-random probes yields no significant fluorescence compared to D514-d$(T)_{12}$. Scale bar: 20 μm. These results suggest labeling stringency of ECHO probes *in vivo*.

Supplementary figure 6. **Redistribution of 28S rRNA in HeLa cells treated with actD and oxaliplatin.** Confocal images of HeLa cells hybridized with D514-28S. Before fixation, HeLa cells were treated for 1 hr with drugs that had previously been shown to trigger morphological changes in nucleoli and in redistribution of 28S rRNA. (A) Vehicle-treated HeLa cells. (B) HeLa cells treated with 50 nM of Actinomycin D or 100 μM of Oxaliplatin. Robust changes in the morphology of 28S rRNA fluorescence foci were detected after the treatments. (C) HeLa cells treated with 400 μM of cisplatin or 50 μM of α-amanitin. In these cells, no apparent morphological changes of 28s rRNA foci were observed. Scale bar: 20 μm. We therefore applied actD and oxaliplatin to treat acute brain slices for their effect on 28S rRNA in cerebellar granule cells.

Supplementary figure 7. **Cell type-dependent foci dynamics of 28S rRNA in response to Oxa and actD.** Cell lines and primary cultures were treated with Oxa (100 μM) or ActD (50 nM) for 60 min,

fixed and hybridized with D514-28S(D) using an ECHO-FISH protocol. (A) *Up,* Representative confocal images of 28S rRNA foci in HeLa, MCF7, SH-SY5Y, hippocampal neurons and cerebellar granule cells before and after Oxa treatment. *Bottom,* Quantification of mean D514 fluorescence intensity at individual foci (MFI) in individual cells. $^{**}P<0.001$. n.s.: not significant. (B) *Up,* Representative confocal images of 28S rRNA foci in different cell types before and after ActD treatment. *Bottom,* Distribution of cell populations with 28S rRNA foci number above mean+SD (purple), equal to mean±SD (chartreuse), or below mean-SD (red) before and after treatment. Imaging parameters, 514 nm excitation, 500-600 nm detection; 0.260 μm x 0.260 μm/pixel; 6 μs pixel dwell times.

Video 1. **Confocal time-lapse images of poly(A) nuclear speckles in living HeLa cells** (also see Fig. 2B)**.** HeLa cells labeled with D514-$(U)_{22}$ were imaged every 800 ms; playback rate is seven frames per second. Poly(A) nuclear speckles were immobile during the 80-sec imaging period.

Video 2. **Confocal time-lapse images of poly(A) RNA with D514-$(U)_{22}$ in cerebellar granule cells** (also see Fig. 7A)**.** Images were acquired at 1 min intervals; playback rate is seven frames per second.

Video 3. **Confocal time-lapse images of U3 snoRNA with D514-U3 in cerebellar granule cells** (also see Fig. 7A)**.** Images were acquired at 1 min intervals; playback rate is seven frames per second.

Video 4. **Confocal time-lapse images of 28S rRNA with D514-28S in cerebellar granule cells** (also see Fig. 7A)**.** Images were acquired at 1 min intervals; playback rate is seven frames per second.

SUPPLEMENTARY REFERENCES

1. Austin, R.J., Xia, T., Ren, J., Takahashi, T.T. and Roberts, R.W. (2002) Designed arginine-rich RNA-binding peptides with picomolar affinity. *J Am Chem Soc*, **124**, 10966-10967.
2. Lim, F. and Peabody, D.S. (1994) Mutations that increase the affinity of a translational repressor for RNA. *Nucleic Acids Res*, **22**, 3748-3752.
3. Strack, R.L., Disney, M.D. and Jaffrey, S.R. (2013) A superfolding Spinach2 reveals the dynamic nature of trinucleotide repeat-containing RNA. *Nat Methods*, **10**, 1219-1224.
4. Turner, B. and Lilley, D.M. (2008) The importance of G.A hydrogen bonding in the metal ion- and protein-induced folding of a kink turn RNA. *J Mol Biol*, **381**, 431-442.

Oomoto et al., Supplemental figure 1

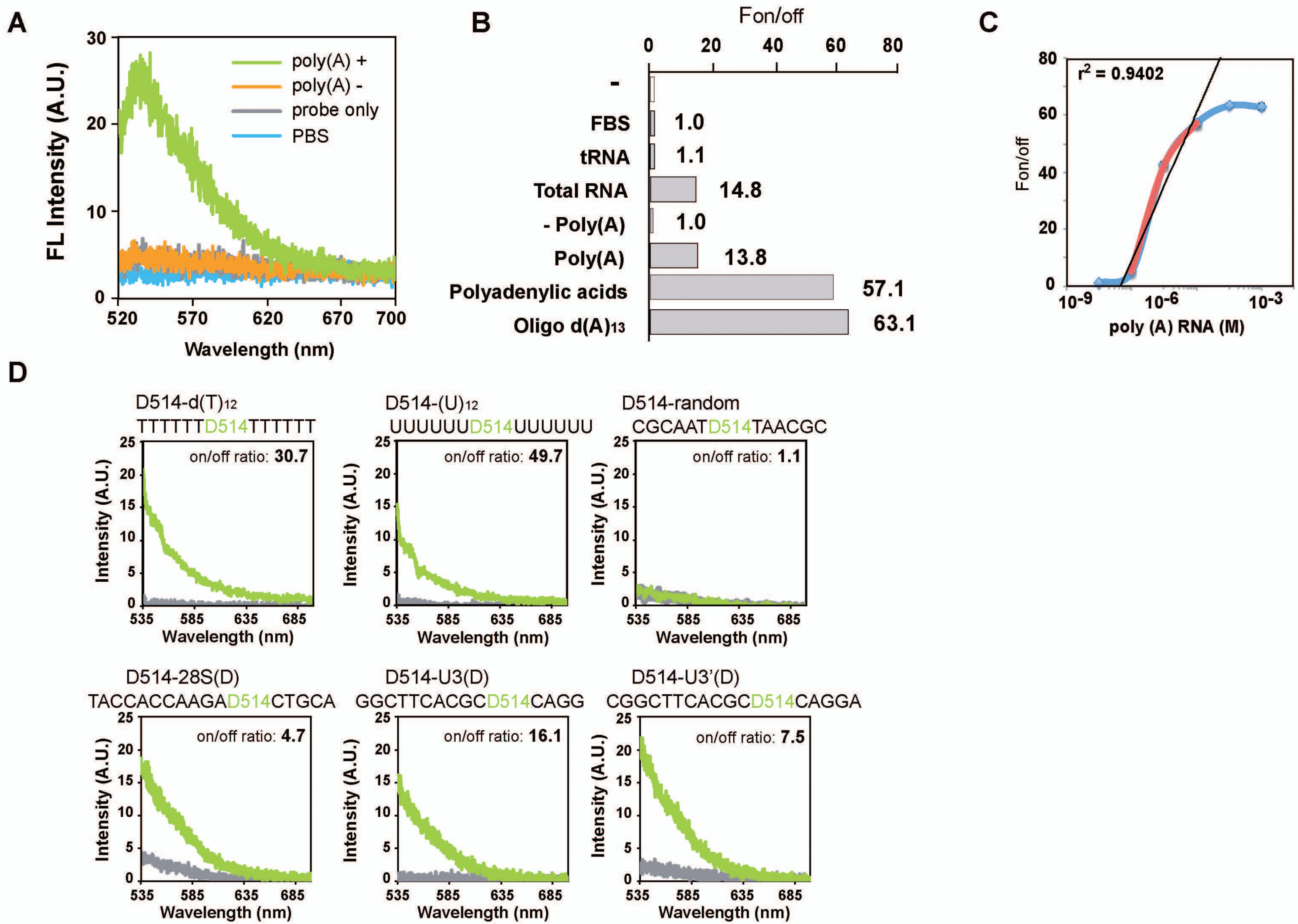

Oomoto et al., Supplementary table 1

	K_D	measurement method	Reference
D514-$d(T)_{12}$	8.54±2.25 μM	Stopped-flow	
D514-U3(D)	5.19±2.61 μM	Stopped-flow	
D514-U3	8.48±4.80 μM	Stopped-flow	
Spinach	450±12 nM	Folding assay	Strack et al., 2013
Spinach2	430±15 nM	Folding assay	Strack et al., 2013
MS2-hairpin/GFP	0.4 nM	Filter-binding assay	Lim and Peabody, 1994
λN22/BoxB	22 nM	Stopped-flow	Austin et al., 2002
L7Ae/Kt-7	10 pM	Stopped-flow	Turner and Lilley, 2008

Oomoto et al., Supplementary figure 2

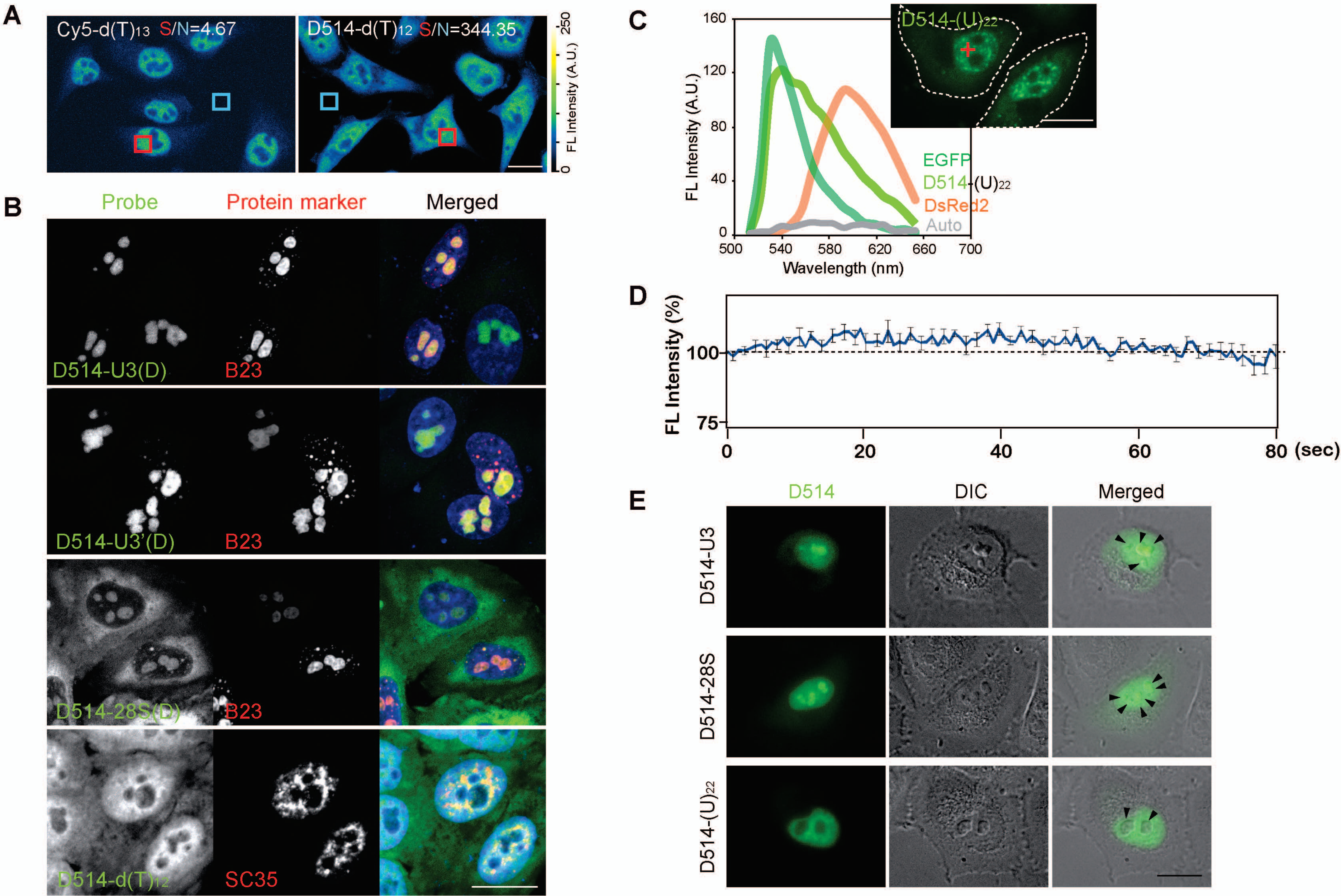

Oomoto et al., Supplementary figure 3

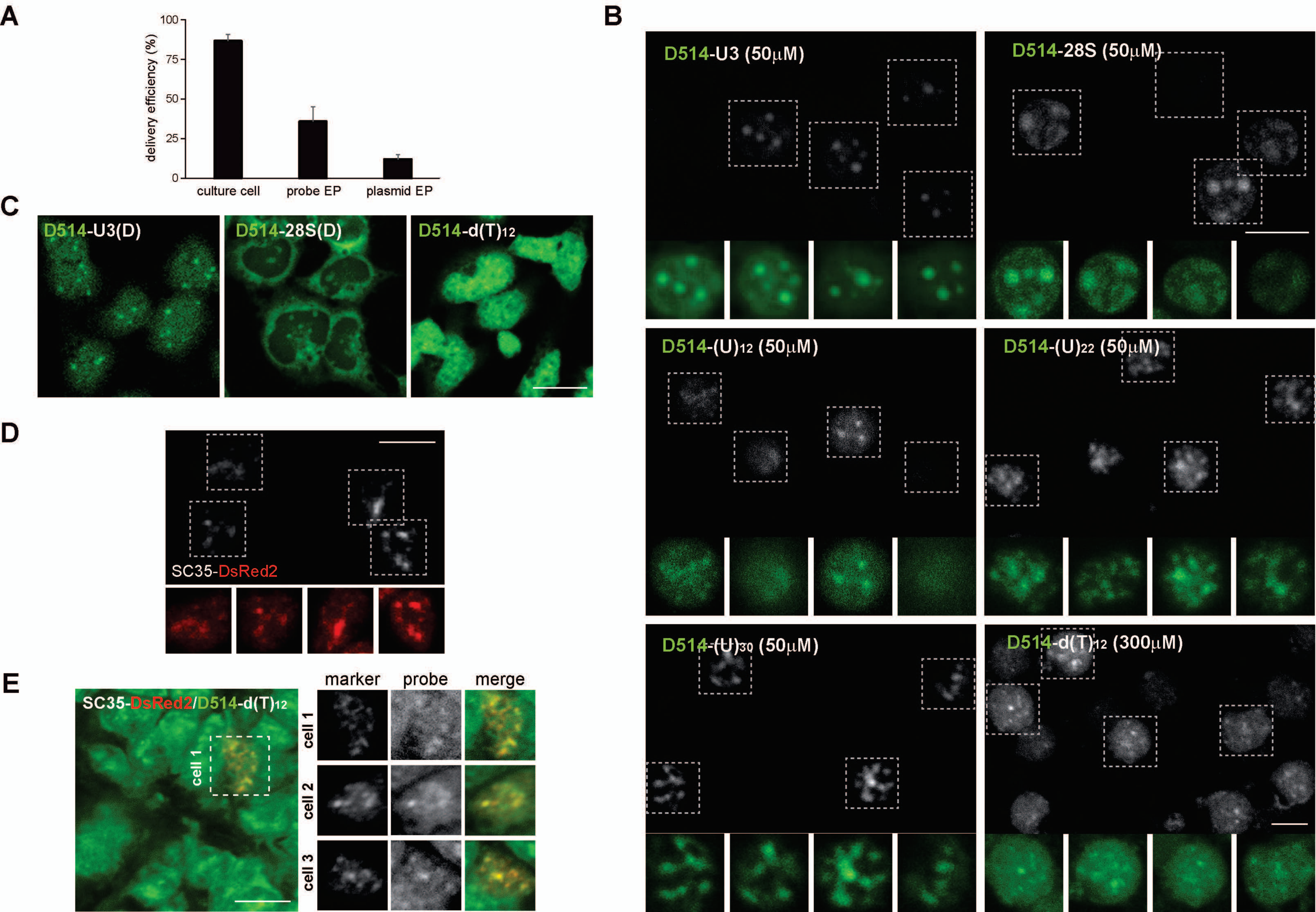

Oomoto et al., Supplementary figure 4

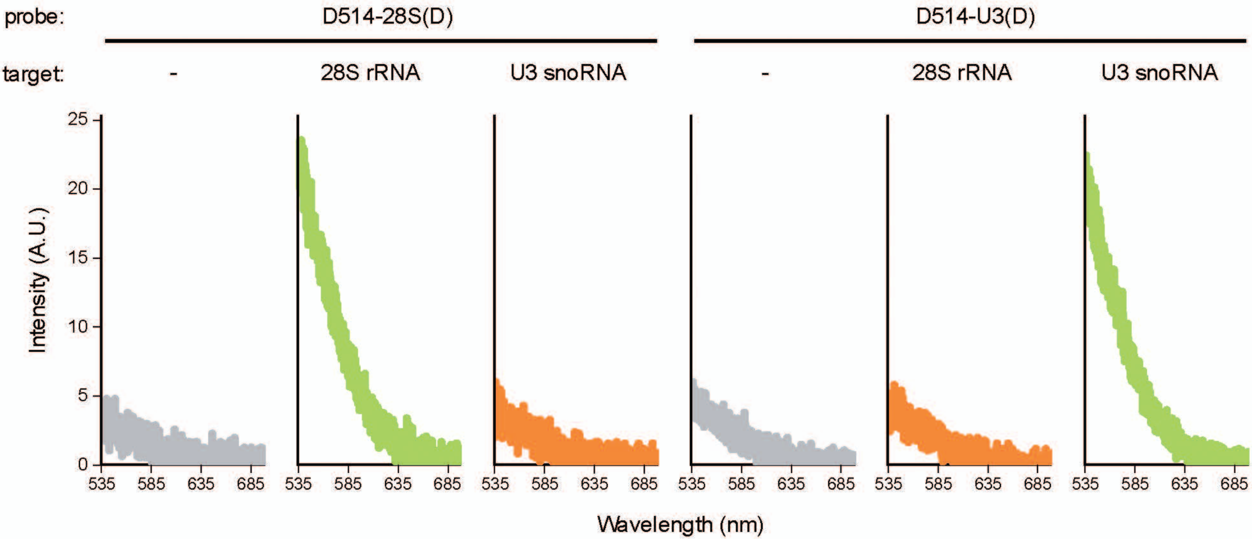

Oomoto et al., Supplementary figure 5

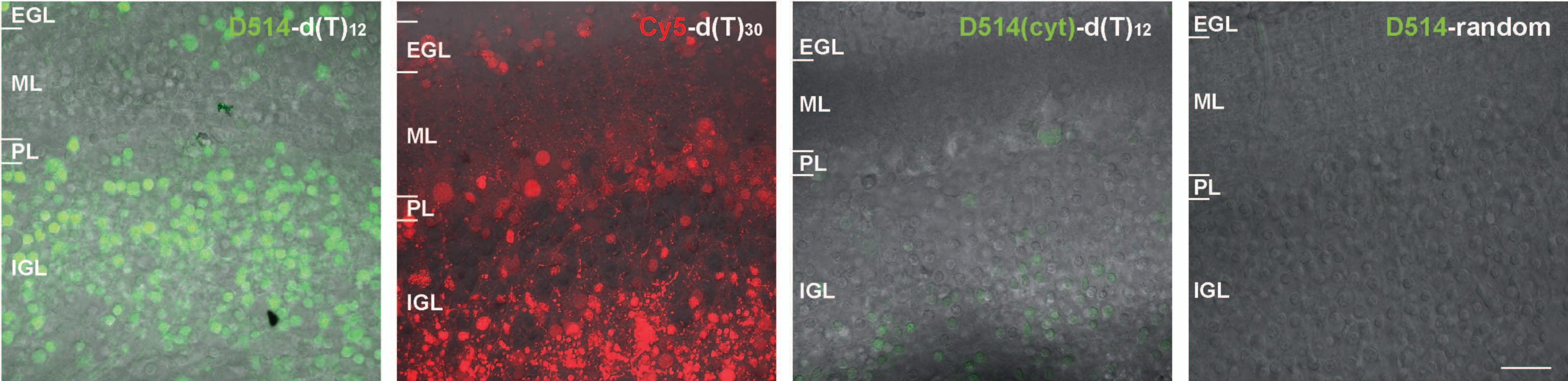

Oomoto et al., Supplementary figure 6

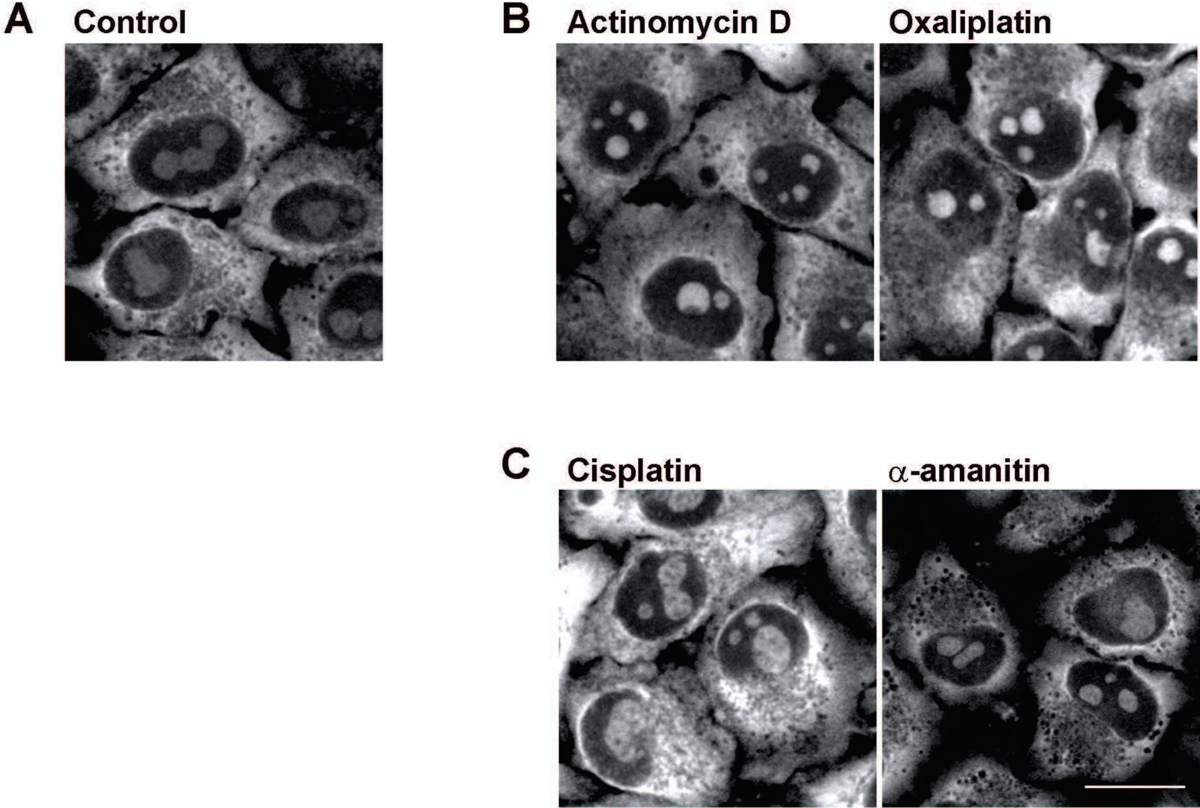

Oomoto et al., Supplementary figure 7

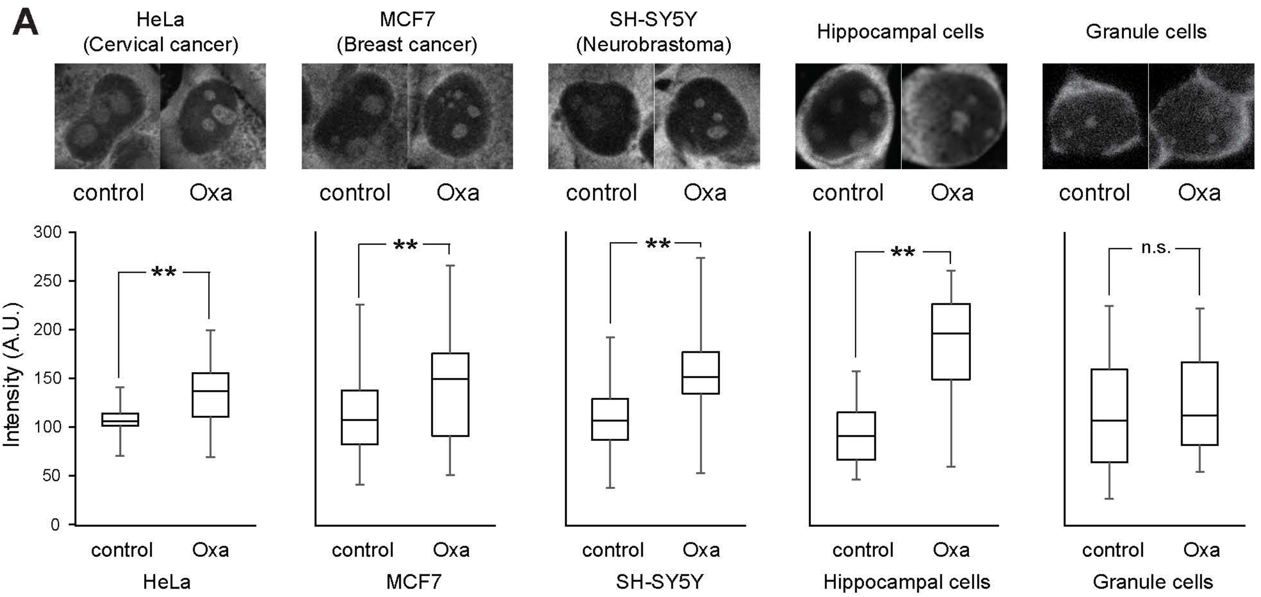

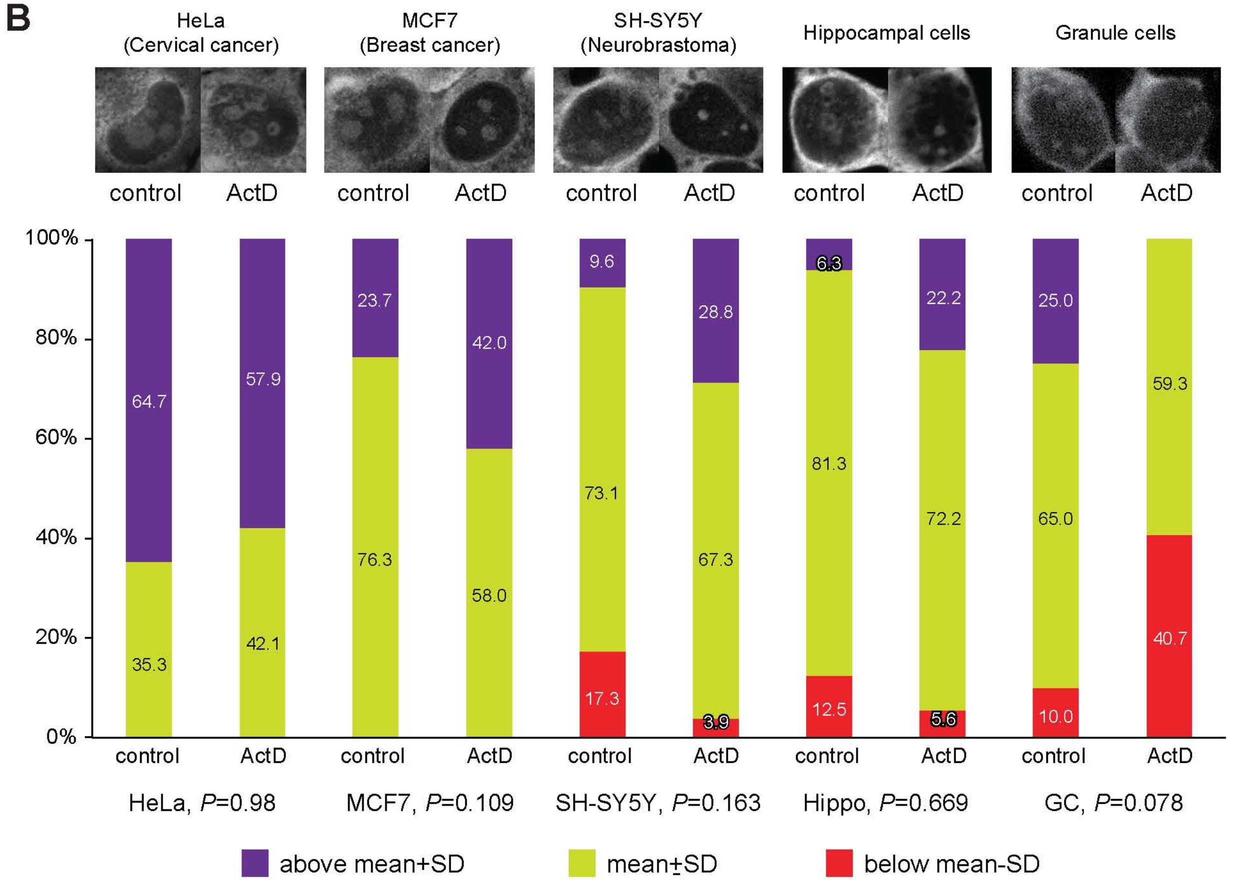

Supplement: SUPPLEMENTARY DATA [file supp_gkv614_oomoto_et_al_supplementary_materials_correct.pdf]
